# The effect of the zero-field splitting in light-induced pulsed dipolar electron paramagnetic resonance (EPR) spectroscopy

**DOI:** 10.5194/mr-4-27-2023

**Published:** 2023-02-08

**Authors:** Andreas Scherer, Berk Yildirim, Malte Drescher

**Affiliations:** Department of Chemistry, Konstanz Research School Chemical Biology, University of Konstanz, 78457 Konstanz, Germany

## Abstract

Laser-induced magnetic dipole (LaserIMD) spectroscopy and light-induced double electron–electron resonance (LiDEER) spectroscopy are important techniques in the emerging field of light-induced pulsed dipolar electron paramagnetic resonance (EPR) spectroscopy (light-induced PDS). These techniques use the photoexcitation of a chromophore to the triplet state and measure its dipolar coupling to a neighboring electron spin, which allows the determination of distance restraints. To date, LaserIMD and LiDEER have been analyzed with software tools that were developed for a pair of two S=1/2 spins and that neglected the zero-field splitting (ZFS) of the excited triplet. Here, we explore the limits of this assumption and show that the ZFS can have a significant effect on the shape of the dipolar trace. For a detailed understanding of the effect of the ZFS, a theoretical description for LaserIMD and LiDEER is derived, taking into account the non-secular terms of the ZFS. Simulations based on this model show that the effect of the ZFS is not that pronounced in LiDEER for experimentally relevant conditions. However, the ZFS leads to an additional decay in the dipolar trace in LaserIMD. This decay is not that pronounced in Q-band but can be quite noticeable for lower magnetic field strengths in X-band. Experimentally recorded LiDEER and LaserIMD data confirm these findings. It is shown that ignoring the ZFS in the data analysis of LaserIMD traces can lead to errors in the obtained modulation depths and background decays. In X-band, it is additionally possible that the obtained distance distribution is plagued by long distance artifacts.

## 1 Introduction

Pulsed dipolar electron paramagnetic resonance (EPR) spectroscopy (PDS) has become an important tool for nanoscale distance determination in soft matter. Its applications include the structural determination of biomacromolecules, like proteins (Yee et al., 2015; Yang et al., 2020; Giannoulis et al., 2020; Weickert et al., 2020; Robotta et al., 2014; Ritsch et al., 2022) DNA (Wojciechowski et al., 2015; Takeda et al., 2004; Marko et al., 2011) and RNA (Collauto et al., 2020), as well as synthetic polymers (Jeschke et al., 2010) and nanoparticles (Hintze et al., 2015; Bücker et al., 2019). PDS measures the dipolar coupling between two spin centers within the molecule under investigation. Oftentimes, the spin centers need to be introduced as spin labels via site-directed labeling, with nitroxide spin probes as the most common example (Hubbell et al., 2013; Roser et al., 2016; García-Rubio, 2020). The most common PDS technique is double electron–electron resonance (DEER, also called PELDOR) spectroscopy (Milov et al., 1981, 1984; Jeschke, 2012). Here, one of the spin labels is excited by microwave pulses at an observer frequency to generate a refocused echo. The excitation of the other spin label by a pump pulse at a second frequency leads to an oscillation of the refocused echo, when the pump pulse is shifted in the time domain. The frequency of this oscillation depends on the inverse cubic distance between the spin labels $r^{-3}$ and, thus, provides distance information for the molecule under investigation (Jeschke, 2012).

**Figure 1:**
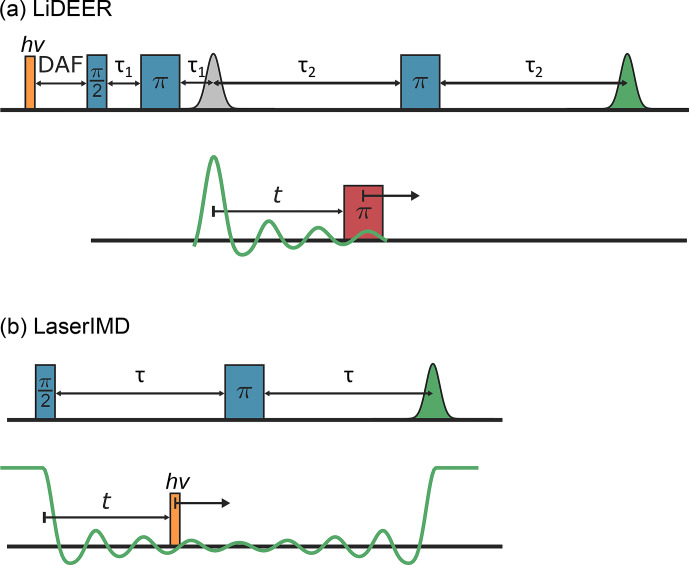
The pulse sequences of **(a)** LiDEER and **(b)** LaserIMD. The observed green echoes are modulated when the pump pulse (LiDEER) or laser flash (LaserIMD) is shifted in the time domain.

The recent years have seen the advent of a new type of spin label that is in an EPR-silent singlet ground state but can be converted transiently to a triplet state by photoexcitation and subsequent intersystem crossing (Di Valentin et al., 2014; Bertran et al., 2022a). In contrast to spin labels with a spin of S=1/2 , like nitroxides, these transient triplet labels are subject to an additional zero-field splitting (ZFS). It is described by the ZFS parameters D and E . By now, several transient triplet labels with different ZFS strengths have been used. Examples are triphenylporphyrin (TPP) ( D=1159 , E=-238 MHz) (Di Valentin et al., 2014), fullerenes ( D=342 , E=-2 MHz) (Wasielewski et al., 1991; Krumkacheva et al., 2019; Timofeev et al., 2022), rose bengal ( D=3671 , E=-319 MHz), eosin Y ( D=2054 , E=-585 MHz), Atto Thio12 ( D=1638 , E=-375 MHz) (Serrer et al., 2019; Williams et al., 2020) and erythrosin B ( D=3486 , E=-328 MHz) (Bertran et al., 2022b). The most common PDS techniques for transient triplet labels are light-induced DEER (LiDEER) and laser-induced magnetic dipole (LaserIMD) spectroscopy (Di Valentin et al., 2014; Hintze et al., 2016). They both allow the determination of distances between one permanent spin label and one transient triplet label. LiDEER is a modification of DEER with an additional laser flash preceding the microwave pulses (see Fig. 1a). The permanent spin is excited by the pump pulse, as it typically has an EPR spectrum that is narrower than the one of the transient triplet label, which gives higher modulation depths. The transient triplet label is observed because, despite its broader EPR spectrum, it is still possible to generate strong echoes, as the photoexcitation of the transient triplet label typically leads to a high spin polarization (Di Valentin et al., 2014). In LaserIMD, on the other hand, the permanent spin label is observed. During the evolution of the observer spin, the transient triplet label is excited by a laser flash (see Fig. 1b). The induced transition from the singlet to the triplet state has an equivalent effect to the microwave pump pulse in DEER and results in an oscillation of the echo of the observer spin. An advantage of LaserIMD is that, in contrast to DEER, the bandwidth of the laser excitation is neither limited by the width of the EPR spectrum of the pump spin nor the resonator bandwidth. This gives virtually infinite excitation bandwidths and promises high modulation depths, even in cases where the microwave excitation bandwidth is smaller than the EPR spectra of the invoked spins (Scherer et al., 2022a).

In previous works, LaserIMD and LiDEER data were analyzed under the assumption that the ZFS of the transient triplet label can be ignored (Di Valentin et al., 2014; Hintze et al., 2016; Bieber et al., 2018; Dal Farra et al., 2019a; Krumkacheva et al., 2019). Under this assumption, the dipolar traces of LaserIMD and LiDEER have the same shape as those of DEER on a label pair with two S=1/2 spins. However, as is shown below, this assumption is only correct if all spin–spin interactions are much smaller than the Zeeman interaction with the external magnetic field. Then, all non-secular terms in the Hamiltonian can be dropped (Manukovsky et al., 2017). The excited triplet state of transient triplet labels with a total spin of S=1 , however, can be subject to a strong ZFS, reaching values of over 1 GHz in many cases (Di Valentin et al., 2014; Williams et al., 2020). For other high-spin labels like Gd ${}^{\mathrm{III}}$ or high-spin Fe ${}^{\mathrm{III}}$ , it is already known that the ZFS can have an effect on the recorded dipolar trace and that it has to be included in the data analysis routine if artifacts in the distance are to be avoided (Maryasov et al., 2006; Dalaloyan et al., 2015; Abdullin et al., 2019).

Here, we set out to investigate the effect of the ZFS in light-induced PDS. Therefore, we are going to derive a theoretical description for light-induced PDS, taking the S=1 spin state and the ZFS of the triplet state into account. Section 3 will report the materials and methods used. In Sect. 4, the theoretical model will be used for numerical simulations of LaserIMD, and time-domain simulations performed for LiDEER will be reported. It will be shown that the effect of the ZFS can result in significant differences in the dipolar traces in both methods compared with the S=1/2 case where the ZFS is ignored; however, this effect is particularly pronounced in LaserIMD. In Sect. 5, experimental LaserIMD and LiDEER traces are shown, and the influence of the ZFS is discussed by comparing the model with the experimental data.

## 2 Theoretical derivation

### 2.1 DEER

For the analysis of DEER data, one typically uses the assumption that both spins are of S=1/2 nature and that the system is in high-field and weak-coupling limit so that all pseudo- and non-secular parts of the spin Hamiltonian can be dropped (Jeschke et al., 2006; Worswick et al., 2018; Fábregas Ibáñez et al., 2020). In this case, there are two coherence transfer pathways that contribute to the DEER signal: one where the pump spin is flipped from the state with $m_{S}=+1/2$ to $m_{S}=-1/2$ and one where it is flipped from $m_{S}=-1/2$ to $m_{S}=+1/2$ . The frequency of the dipolar oscillation of the refocused echo for the two coherence transfer pathways is as follows:

1ωDEER,+12→-12=3cos⁡βdip2-1ωdip,2ωDEER,-12→+12=-3cos⁡βdip2-1ωdip.

Here, $\beta_{\mathrm{dip}}$ is the angle between the dipolar coupling vector and the external magnetic field, and $\omega_{\mathrm{dip}}$ is the dipolar coupling in radial frequency units. $\omega_{\mathrm{dip}}$ depends on the distance r between the two labels:

3 $$\omega_{\mathrm{dip}}=\frac{\mu_{B}^{2}g_{1}g_{2}}{}\frac{1}{r^{3}},$$

with the Bohr magneton $\mu_{B}$ , the reduced Plank constant 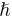 and the g values ( $g_{1}$ and $g_{2}$ ) of the two spin labels. In experiments, one typically measures powder samples; thus, molecules with all orientations with respect to the external field contribute to the signal, and the weighted integral over all angles $\beta_{\mathrm{dip}}$ must be taken (Pake, 1948; Milov et al., 1998). In the high-temperature limit, which is often fulfilled in experiments, the population of the spin states with $m_{S}=+1/2$ to $m_{S}=-1/2$ is virtually identical; therefore, both coherence transfer pathways contribute equally to the signal (Marko et al., 2013). In this case, the integral over all orientations is as follows:

4 $$\begin{array}{rl} S_{\mathrm{DEER}}\left(t,r\right)= & \int_{0}^{\pi /2}d\beta_{\mathrm{dip}}\sin\left(\beta_{\mathrm{dip}}\right)\\ & \mathrm{cos}\left(t\left(3\mathrm{cos}{\left(\beta_{\mathrm{dip}}\right)}^{2}-1\right)\omega_{\mathrm{dip}}(r)\right). \end{array}$$

Here, t is the time at which the pump pulse flips the pump spins. Due to a limited excitation bandwidth and pulse imperfections, not all spins can be excited by the pump pulse; therefore, a part of the signal is not modulated:

5 $$F_{\mathrm{DEER}}\left(t,r\right)=\lambda S_{\mathrm{DEER}}\left(t,r\right)+\left(1-\lambda \right),$$

where the modulation depth λ depends on the fraction of excited pump spins. The experimental signal is the product of this intramolecular contribution $F_{\mathrm{DEER}}\left(t,r\right)$ and a contribution from the intermolecular dipolar interactions B(t) , which is typically termed background. Finally, the contributions from all distances need to be included by integrating over the distance distribution P(r) :

6 $$\begin{array}{rl} V_{\mathrm{DEER}}\left(t\right) & =\int drK_{\mathrm{DEER}}(t,r)P\left(r\right)\\ & =\int drB(t)F_{\mathrm{DEER}}\left(t,r\right)P\left(r\right). \end{array}$$

The kernel $K_{\mathrm{DEER}}(t,r)$ describes the relation between the distance distribution and the measured dipolar trace in DEER. In a sample with a homogenous distribution of spins, the background function can be obtained by integrating over all dipolar interactions within the sample, which results in the following (Hu and Hartmann, 1974):

7 $$B\left(t\right)=\mathrm{exp}\left(-k\left|t\right|\right).$$

The decay constant k is proportional to the spin concentration and modulation depth (Hu and Hartmann, 1974). By inverting Eq. (6), it is possible to extract the distance distribution P(r) from the experimentally recorded signal $V_{\mathrm{DEER}}(t)$ . Because this is an ill-posed problem, this is typically done by advanced techniques like Tikhonov regularization (Bowman et al., 2004; Jeschke et al., 2004) or neural networks (Worswick et al., 2018; Keeley et al., 2022).

### 2.2 LaserIMD

In LaserIMD, the spin system consists of a permanent spin label, which serves as an observer spin, and a transient triplet label, which is excited by a laser flash. In many cases, the permanent spin label is or can be assumed to be a doublet with $S_{D}=1/2$ . Before the photoexcitation, the transient label is still in its singlet state; therefore it interacts with neither the external field B nor the doublet $S_{D}$ . Thus, the Hamiltonian only contains the Zeeman interaction of $S_{D}$ :

8 $${\hat{H}}_{\mathrm{dark}}=2\pi \nu_{D}{\hat{S}}_{D,z}.$$

Here the Zeeman frequency $\nu_{D}=\frac{g_{D}\mu_{B}}{2\pi }B$ , where $g_{D}$ denotes the g values of $S_{D}$ , which is assumed to be isotropic. The Hamiltonian is written in units of radial frequencies. This Hamiltonian has two eigenvalues:

9E+12,dark=2πνD2,10E-12,dark=-2πνD2.

When the laser flash excites the transient triplet label to the triplet state $S_{T}=1$ , the Zeeman interaction of $\;S_{T}$ , the ZFS between the two unpaired electrons that form the triplet $S_{T}$ , and the dipolar coupling between $S_{D}$ and $S_{T}$ has to be included in the Hamiltonian:

11 $$\hat{H}=2\pi \nu_{D}{\hat{S}}_{D,z}+2\pi \nu_{T}{\hat{S}}_{T,z}+S_{T}\cdot D\cdot S_{T}+S_{T}\cdot T\cdot S_{D}.$$

Here, $\nu_{T}=\frac{g_{T}\mu_{B}B}{2\pi }$ is the Zeeman frequency of the spin $S_{T}$ with its isotropic g value ( $g_{T}$ ). $S_{D}$ and $S_{T}$ represent the vectors of the Cartesian spin operators $S_{D}={\left({\hat{S}}_{D,x},\;{\hat{S}}_{D,y},\;{\hat{S}}_{D,z}\right)}^{T}$ and $S_{T}={\left({\hat{S}}_{T,x},\;{\hat{S}}_{T,y},\;{\hat{S}}_{T,z}\right)}^{T}$ . The ZFS tensor D is described by the ZFS values $D=\frac{3}{2}D_{z}$ and $E=\frac{D_{x}-D_{y}}{2}$ , where $D_{x}$ , $D_{y}$ and $D_{z}$ are the eigenvalues of the ZFS tensor (Telser, 2017). Its orientation is described by the three Euler angles, $\alpha_{T}$ , $\beta_{T}$ and $\gamma_{T}$ , that connect the laboratory frame with the molecular frame of the transient triplet label. In the point-dipole approximation, the dipolar coupling tensor T is axial with the eigenvalues $T_{x}=T_{y}=-\omega_{\mathrm{dip}}$ and $T_{z}=2\omega_{\mathrm{dip}}$ (Schweiger and Jeschke, 2001). Its orientation towards the external magnetic field is described by the angle $\beta_{\mathrm{dip}}$ . In the high-field and weak-coupling limit all non- and pseudo-secular terms can be dropped from the Hamiltonian. The remaining secular Hamiltonian (see Eq. S2 in Supplement S1) is already diagonal in the high-field basis with the energy levels $E_{m_{D},\;m_{T}}^{\sec}$ , where $m_{D}$ and $m_{T}$ are the magnetic quantum numbers of the doublet $S_{D}$ and the triplet $S_{T}$ , respectively. The exact expressions for the energies $E_{m_{D},m_{T}}^{\sec}$ can be found in Eqs. (S4)–(S9) in Supplement S1. In LaserIMD, the initial $\frac{\pi }{2}$ -pulse generates a coherence of the observer spin $S_{D}$ . Before the laser excitation, the coherence evolves with a frequency of $E_{+\frac{1}{2},\mathrm{dark}}-E_{-\frac{1}{2},\mathrm{dark}}=2\pi \nu_{D}$ ; it is not influenced by the dipolar coupling because the transient triplet label is still in a singlet state with $S_{T}=0$ and $m_{T}=0$ . The excitation of the transient triplet label leads to three different coherence transfer pathways, depending on which manifold, $m_{T}=1$ , 0 or -1 , of the triplet the transient label is excited to. Depending on the triplet state $m_{T}$ , the coherence will then continue to evolve with $E_{+\frac{1}{2},m_{T}}^{\sec}-E_{-\frac{1}{2},m_{T}}^{\sec}$ . The refocusing π -pulse generates an echo at the time 2τ . Due to the different frequencies before and after the excitation at a variable time t , the coherences are not completely refocused; however, depending on the time of the laser flash, they will have gained a phase $\phi =\omega_{m_{T}}^{\sec}t$ , which depends on the LaserIMD frequency $\omega_{m_{T}}^{\sec}$ of the corresponding triplet manifold $m_{T}$ . When only the secular terms are considered in the Hamiltonian, the LaserIMD frequencies $\omega_{m_{T}}^{\sec}$ do not depend on the ZFS, as its secular terms cancel each other out, and the same expressions as those of Hintze et al. (2016) are obtained:

12ω+1sec=E+12,+1sec-E-12,+1sec-E+12,dark-E-12,dark=3cos⁡βdip2-1ωdip,13ω0sec=E+12,0sec-E-12,0sec-E+12,dark-E-12,dark=0,14ω-1sec=E+12,-1sec-E-12,-1sec-E+12,dark-E-12,dark=-3cos⁡βdip2-1ωdip.

When the transient triplet label is excited to $m_{T}=1$ or $m_{T}=-1$ , the LaserIMD frequencies in secular approximation from Eqs. (12) and (14) are identical to the DEER frequencies in Eqs. (1) and (2), respectively. Here, the laser flash leads to a change in the magnetic quantum number of $\Delta m_{T}=\pm 1$ , which is equivalent to the effect of the microwave pump pulse in DEER. In the case when the transient triplet label is excited to the state $m_{T}=0$ , however, the secular approximation predicts that the echo is not oscillating, as – loosely speaking – there is no change in the magnetic spin quantum number of the transient triplet label, which means that the dipolar coupling is not changed. As is the case in DEER, the measured signal is the average over all orientations of the spin system. Whereas it is only necessary to consider the orientation of the dipolar vector in DEER, the orientation of the transient triplet label must also be taken into account in LaserIMD; therefore, it is necessary to also integrate over the three corresponding Euler angles $\alpha_{T}$ , $\beta_{T}$ and $\gamma_{T}$ (Bak and Nielsen, 1997). In the absence of orientation selection, the orientation of the dipolar vector and the transient triplet label are not correlated, and the integration over the corresponding Euler angles can be done independently. This is often realized in practical applications where flexible linkers are used to attach labels to the studied molecule. As the triplet state of the transient label is reached by intersystem crossing, the population of the three high-field triplet states, $m_{T}=+1$ , 0, -1 , depends on the orientation of the transient label with respect to the external magnetic field and the populations $P_{x}$ , $P_{y}$ and $P_{z}$ of the zero-field eigenstates (Rose, 1995). The contribution of the three coherence transfer pathways must be weighted by population of these high-field states; this gives (still in secular approximation) the following three expressions:

15S+1sect,r=18π2∫02πdαT∫0πdβTsin⁡βT∫02πdγT(Pz2sin⁡2βT+Px2cos⁡2βT+sin⁡2βTsin⁡2γT+Py2cos⁡2βT+sin⁡2βTcos⁡2γT)∫0π2dβdipsin⁡βdipexp⁡-iω+1secβdipt,16S0sect,r=18π2∫02πdαT∫0πdβTsin⁡βT∫02πdγT(Pzcos⁡2βT+Pxsin⁡2βTcos⁡2γT+Pysin⁡2βTsin⁡2γT)∫0π/2dβdipsin⁡βdipexp⁡(-iω0secβdipt),

17 $$\begin{array}{rl} S_{-1}^{\sec}\left(t,r\right)= & \frac{1}{8\pi^{2}}\int_{0}^{2\pi }d\alpha_{T}\int_{0}^{\pi }d\beta_{T}\mathrm{sin}\left(\beta_{T}\right)\int_{0}^{2\pi }\\ & d\gamma_{T}(\frac{P_{z}}{2}{\mathrm{sin}}^{2}\left(\beta_{T}\right)+\frac{P_{x}}{2}\\ & \left({\mathrm{cos}}^{2}\left(\beta_{T}\right)+{\mathrm{sin}}^{2}\left(\beta_{T}\right){\mathrm{sin}}^{2}\left(\gamma_{T}\right)\right)\\ & +\frac{P_{y}}{2}\left({\mathrm{cos}}^{2}\left(\beta_{T}\right)+{\mathrm{sin}}^{2}\left(\beta_{T}\right){\mathrm{cos}}^{2}\left(\gamma_{T}\right)\right))\\ & \int_{0}^{\frac{\pi }{2}}d\beta_{\mathrm{dip}}\mathrm{sin}\left(\beta_{\mathrm{dip}}\right)\mathrm{exp}\left(-i\omega_{-1}^{\sec}\left(\;\beta_{\mathrm{dip}}\right)t\right). \end{array}$$

Performing the integration over the orientations of the transient label $\alpha_{T}$ , $\beta_{T}$ and $\gamma_{T}$ and taking the sum gives the following expression (Williams et al., 2020):

18 $$\begin{array}{rl} S_{\mathrm{LaserIMD}}^{\sec}\left(t,r\right) & =S_{+1}^{\sec}\left(t,r\right)+S_{0}^{\sec}\left(t,r\right)+S_{-1}^{\sec}\left(t,r\right)=\frac{2}{3}\\ & \;\;\;\int_{0}^{\pi /2}\mathrm{cos}\left(\omega_{\mathrm{dip}}\left(3{\mathrm{cos}}^{2}\left(\beta_{\mathrm{dip}}\right)-1\right)t\right)\\ & \;\;\;\mathrm{sin}(\beta_{\mathrm{dip}})d\beta_{\mathrm{dip}}+\frac{1}{3}\\ & =\frac{2}{3}\;S_{\mathrm{DEER}}\left(t,r\right)+\frac{1}{3}. \end{array}$$

In secular approximation, the first term of the LaserIMD signal is equivalent to the trace $S_{\mathrm{DEER}}\left(t\right)$ (Edwards and Stoll, 2018). The second term is an additional non-modulated contribution. For the final expression for the kernel $K_{\mathrm{LaserIMD}}^{\sec}(t,r)$ , the quantum yield of the triplet state is considered by an additional factor γ , and the intermolecular interaction to other spins in the sample has to be considered as background B(t) :

19 $$K_{\mathrm{LaserIMD}}^{\sec}\left(t,r\right)=B\left(t\right)\left(\gamma S_{\mathrm{LaserIMD}}^{\sec}\left(t,r\right)+1-\gamma \right).$$

This can be rewritten as follows:

20 $$K_{\mathrm{LaserIMD}}^{\sec}\left(t,r\right)=B\left(t\right)\left(\lambda S_{\mathrm{DEER}}\left(t,r\right)+1-\lambda \right),$$

with the modulation depth λ=2/3γ . The only difference between LaserIMD in the secular approximation and DEER is that in LaserIMD, even for a triplet yield of γ=100% , there is coherence transfer pathway with $\Delta m_{S}=0$ that does not result in a dipolar oscillation, which limits the maximum achievable modulation depth to $66.\bar{6}\;\%$ . The calculations so far show that, if the secular approximation can be employed, the ZFS has no effect on the LaserIMD trace, and it is possible to analyze experimentally recorded LaserIMD data with the same kernel that can be used for DEER.

**Figure 2:**
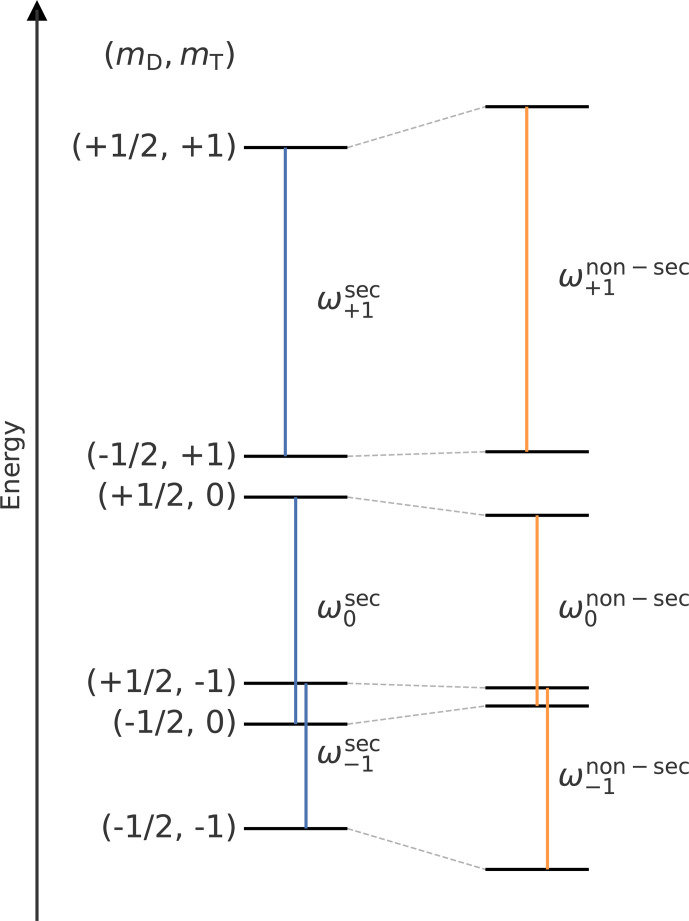
Energy level diagram (not to scale) after the transient triplet label has been excited to the triplet state demonstrating the shift that is induced by the non-secular terms of the ZFS and dipolar coupling from Eq. (S3). The energy levels in secular approximation are shown on the left, and the levels with the non-secular terms are shown on the right. The vertical lines in blue (secular approximation) and orange (non-secular terms included) indicate the coherences of the permanent spin label that are excited during the LaserIMD pulse sequence. They are marked with the corresponding transition frequencies.

Even though the ZFS has no effect in the secular approximation in LaserIMD, it cannot be taken for granted that the non-secular terms can be ignored because the ZFS of some transient triplet labels can be quite large (Williams et al., 2020). Here, we additionally consider the terms ${\hat{S}}_{T,z}{\hat{S}}_{T,+}+{\hat{S}}_{T,+}{\hat{S}}_{T,z}$ and ${\hat{S}}_{T,-}{\hat{S}}_{T,z}+{\hat{S}}_{T,-}{\hat{S}}_{T,z}$ from the ZFS interaction and the terms ${\hat{S}}_{D,z}{\hat{S}}_{T,+}$ and ${\hat{S}}_{D,z}{\hat{S}}_{T,-}$ from the dipolar coupling. They connect the adjacent triplet states |+1〉 and |0〉 and |0〉 and |-1〉 of the triplet manifold and shift their energy in second order (Hagston and Holmes, 1980). This is illustrated in Fig. 2. The details of this calculation are described in Supplement S1. For this calculation, the remaining ZFS terms ${\hat{S}}_{T,+}^{2}$ and ${\hat{S}}_{T,-}^{2}$ were ignored. They connect the triplet states |+1〉 and |-1〉 , which have a larger energy difference than adjacent states. Therefore, the second-order energy shift caused by ${\hat{S}}_{T,+}^{2}$ and ${\hat{S}}_{T,-}^{2}$ is weaker than those of the considered terms. The terms ${\hat{S}}_{D,+}{\hat{S}}_{T,+}$ ; ${\hat{S}}_{D,-}{\hat{S}}_{T,+}$ ; ${\hat{S}}_{D,+}{\hat{S}}_{T,-}$ ; ${\hat{S}}_{D,-}{\hat{S}}_{T,-}$ ; ${\hat{S}}_{D,+}{\hat{S}}_{T,z}$ ; and ${\hat{S}}_{D,-}{\hat{S}}_{T,z}$ of the dipolar coupling were also ignored. They connect the spin states of different manifolds of the doublet spin, and the corresponding energies cannot be significantly shifted by the comparably weak dipolar coupling. It is shown in Supplement S2 that the included non-secular terms from Eq. (S3) are sufficient at the magnetic field strengths that are relevant for experimental conditions, and no further distortions are to be expected due to the omitted terms.

The shift in the energy levels also leads to a shift in the LaserIMD frequencies (see Supplement S1):

21ω+1non-sec=E+12,+1non-sec-E-12,+1non-sec-E+12,dark-E-12,dark=3cos⁡βdip2-1+δZFSsin⁡2βdipωdip,22ω0non-sec=E+12,0non-sec-E-12,0non-sec-E+12,dark-E-12,dark=-2δZFSsin⁡2βdipωdip,23ω-1non-sec=E+12,-1non-sec-E-12,-1non-sec-E+12,dark-E-12,dark=-3cos⁡βdip2-1+δZFSsin⁡2βdipωdip,

where

24 $$\delta_{\mathrm{ZFS}}=\frac{\begin{array}{c} 3\mathrm{sin}\left(2\beta_{T}\right)\mathrm{cos}\left(\alpha_{T}\right)D\\ -6\sin\left(\beta_{T}\right)\left(\mathrm{cos}\left(\beta_{T}\right)\mathrm{cos}\left(2\gamma_{T}\right)\mathrm{cos}\left(\alpha_{T}\right)\right.\\ \left.-\sin\left(2\gamma_{T}\right)\mathrm{sin}\left(\alpha_{T}\right)\right)E \end{array}}{8\pi \nu_{T}}.$$

As can be seen from Eqs. (21)–(23), the frequencies $\omega_{+1}^{\text{non-sec}}$ and $\omega_{-1}^{\text{non-sec}}$ are the sum of the unperturbed frequencies $\omega_{+1}^{\sec}$ and $\omega_{-1}^{\sec}$ and a frequency shift $\delta_{\mathrm{ZFS}}\mathrm{sin}\left(2\beta_{\mathrm{dip}}\right)\omega_{\mathrm{dip}}$ , which contains the effect of the ZFS. Most notably, the coherence transfer pathway with $\Delta m_{T}=0$ does not lead to a vanishing LaserIMD frequency, as was the case in the secular approximation. Instead, we find that $\omega_{0}^{\text{non-sec}}$ equals twice the negative of the frequency shift that is experienced by the other two coherence transfer pathways. The frequency shift scales with $\delta_{\mathrm{ZFS}}$ , which depends on the ZFS values D and E , the Zeeman frequency of the transient triplet label $\omega_{T}$ , and the orientation of the transient triplet label, described by $\alpha_{T}$ , $\beta_{T}$ and $\gamma_{T}$ . At a higher ZFS and a smaller magnetic field, the shift in the LaserIMD frequencies will be larger, so that larger disturbances in the LaserIMD trace can be expected in these cases.

The powder average is more complex when the non-secular terms are included, as the LaserIMD frequencies now also depend on the orientation of the transient triplet label. Still assuming no orientation selection, this gives the following integrals:

25S+1non-sect,r=18π2∫02πdαT∫0πdβtsin⁡βT∫02πdγT(Pz2sin⁡2βT+Px2cos⁡2βT+sin⁡2βTsin⁡2γT+Py2cos⁡2βT+sin⁡2βTcos⁡2γT)∫0π2dβdipsin⁡βdipexp⁡-iω+1non-secαT,βT,γT,βdipt,26S0non-sect,r=18π2∫02πdαT∫0πdβTsin⁡βT∫02πdγT(Pzcos⁡2βT+Pxsin⁡2βTcos⁡2γT+Pysin⁡2βTsin⁡2γT)∫0π/2dβdipsin⁡βdipexp⁡-iω0non-secαT,βT,γT,βdipt,27S-1non-sect,r=18π2∫02πdαT∫0πdβTsin⁡βt∫02πdγT(Pz2sin⁡2βT+Px2cos⁡2βT+sin⁡2βTsin⁡2γT+Py2cos⁡2βT+sin⁡2βTcos⁡2γT)∫0π2dβdipsin⁡βdipexp⁡-iω-1non-secαT,βT,γT,βdipt.

The sum over these terms gives the final intramolecular contribution in LaserIMD:

28 $$S_{\mathrm{LaserIMD}}^{\text{non-sec}}(t)=S_{+1}^{\text{non-sec}}(t)+S_{0}^{\text{non-sec}}(t)+S_{-1}^{\text{non-sec}}(t).$$

By including incomplete excitation and the intermolecular dipolar interactions, one arrives at the final model:

29 $$K_{\mathrm{LaserIMD}}^{\text{non-sec}}\left(t,r\right)=B\left(t\right)\left(\lambda S_{\mathrm{LaserIMD}}^{\text{non-sec}}\left(t,r\right)+1-\lambda \right).$$

Unlike the case for the secular approximation, the integrals are difficult to solve analytically, and further insight into this expression will be gained by numerical integrations in the next sections. However, it can already be seen without further calculations that, with the non-secular terms, the ZFS has an influence in LaserIMD and that the resulting kernel no longer corresponds to the kernel $K_{\mathrm{DEER}}\left(t,r\right)$ of the S=1/2 case.

### 2.3 LiDEER

In LiDEER, the transient triplet label is observed, and the permanent spin label is pumped. For simplicity, we will derive the expressions within the secular approximation first and afterwards turn to the case that includes the non-secular terms. Due to the limited excitation bandwidth of the observer pulse, either the transition between the states with $m_{T}=1$ and $m_{T}=0$ or the states with $m_{T}=0$ and $m_{T}=-1$ of the transient triplet label is excited. If the transition between the states $m_{T}=1$ and $m_{T}=0$ is excited, the excited coherence of the triplet spin will either evolve with the frequency $\omega_{+\frac{1}{2},\;1↔0}^{\sec}=E_{+\frac{1}{2},+1}^{\sec}-E_{+\frac{1}{2},0}^{\sec}$ or $\omega_{-\frac{1}{2},\;1↔0}^{\sec}=E_{-\frac{1}{2},+1}^{\sec}-E_{-\frac{1}{2},0}^{\sec}$ , depending on whether the permanent spin label is in the state with $m_{D}=1/2$ or $m_{D}=-1/2$ . Pumping the permanent spin label at the time t will result in a transition from $m_{D}=+\frac{1}{2}$ to $m_{D}=-\frac{1}{2}$ (or vice versa), and the frequency $\omega_{+\frac{1}{2},\;1↔0}^{\sec}$ or $\omega_{-\frac{1}{2},\;1↔0}^{\sec}$ with which the coherence evolves will change accordingly. At the time of the echo, the coherence will have gained a phase $\phi =\omega_{\pm \frac{1}{2}\to \mp \frac{1}{2},\;\;+1↔0}^{\sec}t$ , where $\omega_{\pm \frac{1}{2}\to \mp \frac{1}{2},+1↔0}^{\sec}$ denotes the LiDEER frequencies of the two coherence transfer pathways:

30ω+12→-12,+1↔0sec=E+12,+1sec-E+12,0sec-E-12,+1sec-E-12,0sec=3cos⁡βdip2-1ωdip,31ω-12→+12,+1↔0sec=E-12,+1sec-E-12,0sec-E+12,0sec-E+12,-1sec=-3cos⁡βdip2-1ωdip.

When the other transition of the triplet spin from $m_{T}=0$ and $m_{T}=-1$ is excited by the observer pulse, the frequencies are the same:

32ω+12→-12,0↔-1sec=E+12,+1sec-E+12,0sec-E-12,+1sec-E-12,0sec=3cos⁡βdip2-1ωdip,33ω-12→+12,0↔-1sec=E-12,+1sec-E-12,0sec-E+12,0sec-E+12,-1sec=-3cos⁡βdip2-1ωdip.

As those are the same frequencies as the ones in DEER with two S=1/2 spins, one eventually arrives at the same kernel $K_{\mathrm{DEER}}(t,r)$ . This means that, as was the case in LaserIMD, the secular terms of the ZFS cancel each other out, and there is no effect of the ZFS on the LiDEER trace. In contrast to LaserIMD in secular approximation, there are also no coherence transfer pathways with $\Delta m_{D}=0$ , so that the maximum achievable modulation depth in LiDEER is 100 %.

It seems obvious that the same non-secular terms that led to change in the LaserIMD frequencies are also relevant in LiDEER. Therefore, the LiDEER frequencies were also determined from the energy levels $E_{m_{D},m_{T}}^{\text{non-sec}}$ that include the effects of the ZFS:

34ω+12→-12,+1↔0non-sec=E+12,+1non-sec-E+12,0non-sec-E-12,+1non-sec-E-12,0non-sec=(3cos⁡βdip2-1+3δZFSsin⁡2βdip)ωdip,35ω-12→+12,+1↔0non-sec=E-12,+1non-sec-E-12,0non-sec-E+12,0non-sec-E+12,-1non-sec=-(3cos⁡βdip2-1+3δZFSsin⁡2βdip)ωdip,36ω+12→-12,0↔-1non-sec=E+12,+1non-sec-E+12,0non-sec-E-12,+1non-sec-E-12,0non-sec=(3cos⁡βdip2-1-3δZFSsin⁡2βdip)ωdip,37ω-12→+12,0↔-1non-sec=E-12,+1non-sec-E-12,0non-sec-E+12,0non-sec-E+12,-1non-sec=-(3cos⁡βdip2-1-3δZFSsin⁡2βdip)ωdip.

It can again be seen that the ZFS leads to a shift in the dipolar frequencies. This shift is, besides the factor of 3, identical to the one that was obtained for the LaserIMD frequencies $\omega_{+1}^{\text{non-sec}}$ and $\omega_{-1}^{\text{non-sec}}$ . From here, the next step is again the averaging over the orientations of the transient triplet label and the dipolar coupling vector that contribute to the LiDEER signal. However, this is even more complicated than it was in LaserIMD, where all orientations are evenly excited by the laser flash. In LiDEER, the triplet spins are also excited by microwave pulses which typically have a bandwidth that is much narrower than the EPR spectrum of the transient triplet label. For example, the frequently used porphyrin labels have an EPR spectrum that is over 2 GHz broad (Di Valentin et al., 2014) of which a typical rectangular microwave pulse with a length of 10 ns can only excite roughly 120 MHz (Schweiger and Jeschke, 2001). Therefore, not all orientations of the transient triplet labels contribute to the LiDEER signal, and it is rather tedious to even derive an expression for the integrals that describe the orientation averaging. To circumvent this problem, the LiDEER traces will be calculated by time-domain simulations with weak microwave pulses in the next sections.

## 3 Materials and methods

### 3.1 Simulations

The powder averages for LaserIMD were performed by a numerical integration of Eqs. (25)–(27) with custom MATLAB (version 2020b) scripts. For the angle $\beta_{\mathrm{dip}}$ , a linear, equidistant grid from 0 to $\frac{\pi }{2}$ was used. Each value was weighted proportional to $\sin(\beta_{\mathrm{dip}})$ . For the orientation of the transient triplet label, a grid with all three Euler angles, $\alpha_{T}$ , $\beta_{T}$ and $\gamma_{T}$ , including the corresponding weights, was calculated according to the REPULSION approach (Bak and Nielsen, 1997; Hogben et al., 2011) with the Spinach (version 2.6.5625) software package (Hogben et al., 2011). To check for a sufficient convergence, a test run with an increasing numbers of points for the two grids was simulated. The test run was stopped when the relative change Δϵ in the simulated signal, when the number of grids points was increased, was below 1 %. For $\beta_{\mathrm{dip}}$ , a grid size of 200 points was sufficient, whereas for $\alpha_{T}$ , $\beta_{T}$ and $\gamma_{T}$ 12 800 points were necessary. For details on the convergence behavior, see Supplement S3.

The time-domain simulations for LiDEER were performed with Spinach version 2.6.5625 (Hogben et al., 2011). The powder averaging was done with the same grids that were used for LaserIMD. For details, see Supplement S8. The source code for the LiDEER simulations can be downloaded from https://github.com/andreas-scherer/LiDEER_simulations.git, last access: 8 January 2023.

### 3.2 Experiments and data analysis

**Figure 3:**
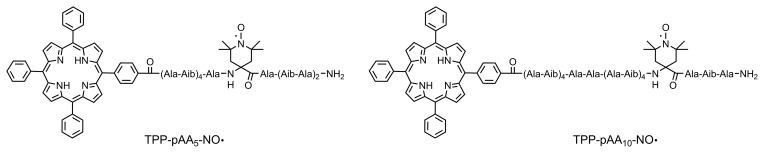
Chemical structures of the peptides TPP–pAA ${}_{5}$ –NO ⚫ and TPP–pAA ${}_{10}$ –NO ⚫ , where the letter code “Ala” denotes l-alanine, and “Aib” denotes α -isobutyric acid.

LaserIMD and LiDEER measurements were performed on the two peptides TPP–pAA ${}_{5}$ –NO ⚫ and TPP–pAA ${}_{10}$ –NO ⚫ shown in Fig. 3. They were purchased from Biosynthan (Berlin) as powder samples and used without further purification. They were dissolved in MeOD / D ${}_{2}$ O ( 98/2 vol %) and, prior to freezing in liquid nitrogen, they were degassed with three freeze–pump–thaw cycles. Light excitation was performed at a wavelength of 510 nm by an Nd : YAG laser system from EKSPLA (Vilnius) that was coupled into the resonator via a laser fiber. EPR measurements were performed on a commercial Bruker ELEXSYS-E580 spectrometer: X-band measurements in an ER4118X-MS3 resonator and Q-band measurements in an ER5106QT-2 resonator. In X-band, the resonator was critically coupled to a Q value of ≈ 900–2000, whereas it was overcoupled to a Q value of ≈ 200 in Q-band. LaserIMD was recorded with the pulse sequence π/2-τ-π-t - laser pulse - ( τ-t ) - echo (Hintze et al., 2016). A two-step phase cycle was implemented for baseline correction. Signal averaging was done by recording 10 shots per point. The zero-time correction was performed by recording a short refocused LaserIMD (reLaserIMD) (Dal Farra et al., 2019a) trace, as reported in Scherer et al. (2022a). LiDEER measurements were performed with the following pulse sequence: laser pulse – DAF – $\pi /2-\tau_{1}-\pi -t-\pi_{\mathrm{pump}}-(\tau_{1}+\tau_{2}-t)-\pi -\tau_{2}-$ echo (Di Valentin et al., 2014). The delay-after-flash (DAF) was set to 500 ns, and $\tau_{1}$ was set to 400 ns. Nuclear modulation averaging was performed by varying the $\tau_{1}$ time in eight steps with $\Delta \tau_{1}=16$ ns. Phase cycling was performed with an eight-step scheme (( x ) [ x ] xp x ), as proposed by Tait and Stoll (2016). The LiDEER data were analyzed with the Python DeerLab (version 0.13.2) software package (Fábregas Ibáñez et al., 2020) and Python 3.9 with the DEER kernel $K_{\mathrm{DEER}}(t,r)$ and Tikhonov regularization. A 3D homogenous background function was used, and the regularization parameter was chosen according to the Akaike information criterion (Edwards and Stoll, 2018). The validation was performed with bootstrapping by analyzing 1000 samples generated with artificial noise. The error was then calculated as the 95 % confidence interval. Further details can be found in Supplement S7 and S10.

## 4 Results and discussion

### 4.1 LaserIMD simulations

**Figure 4:**
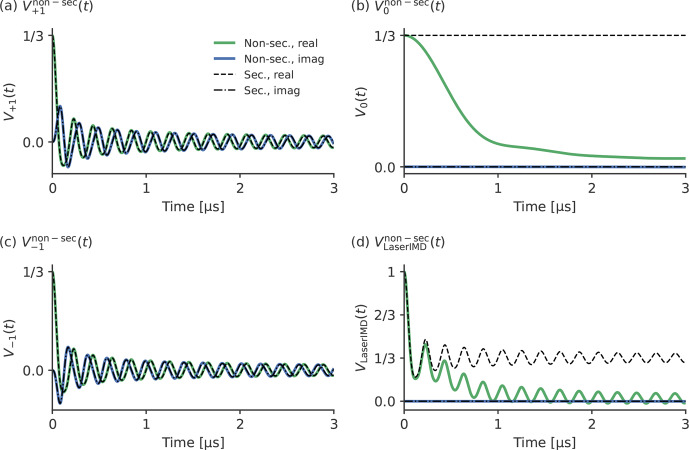
Comparison of simulated LaserIMD traces with and without non-secular interactions with the values D=1159 and E=-238 MHz; $P_{x}=0.33$ , $P_{y}=0.41$ and $P_{z}=0.26$ ; $\nu_{T}=9.3$ GHz (X-band); and r=2.2 nm for **(a)** $V_{+1}^{\text{non-sec}}\left(t\right)$ , **(b)** $V_{0}^{\text{non-sec}}\left(t\right)$ , **(c)** $V_{-1}^{\text{non-sec}}\left(t\right)$ and **(d)** $V_{\mathrm{LaserIMD}}^{\text{non-sec}}\left(t\right)=V_{+1}^{\text{non-sec}}\left(t\right)+V_{0}^{\text{non-sec}}\left(t\right)+V_{-1}^{\text{non-sec}}\left(t\right)$ .

An initial simulation to study the effect of the ZFS in LaserIMD was performed for X-band ( $\nu_{T}=9.3\mathrm{GHz})$ with a dipolar coupling that corresponds to a distance of r=2.2 nm, a ZFS of D=1159 and E=-238 MHz, and zero-field populations of $P_{x}=0.33$ , $P_{y}=0.41$ and $P_{z}=0.26$ . The ZFS and zero-field populations correspond to TPP, which is often used to perform LaserIMD and LiDEER measurements (Di Valentin et al., 2014; Hintze et al., 2016; Di Valentin et al., 2016; Bieber et al., 2018; Bertran et al., 2020). For simplicity, a complete excitation of the transient triplet label ( γ=1 ) was assumed, and no background was added ( B(t)=1) . For a more detailed analysis, the contributions from the three coherence transfer pathways with $\Delta m_{T}=1,\;0,\;\;-1$ , termed $V_{+1}^{\text{non-sec}}\left(t\right)$ , $V_{0}^{\text{non-sec}}(t)$ and $V_{-1}^{\text{non-sec}}\left(t\right)$ , respectively, are simulated separately and presented in Fig. 4 with their resulting sum $V_{\mathrm{LaserIMD}}^{\text{non-sec}}(t)$ . They are also compared with the corresponding traces from the secular approximation, $V_{\mathrm{LaserIMD}}^{\sec}(t)$ , $V_{+1}^{\sec}(t)$ , $V_{0}^{\sec}(t)$ and $V_{-1}^{\sec}(t)$ , where the ZFS is ignored. The comparison of the traces including and excluding the ZFS ( $V_{+1}^{\text{non-sec}}\left(t\right)$ and $V_{-1}^{\text{non-sec}}\left(t\right)$ with $V_{+1}^{\sec}\left(t\right)$ and $V_{-1}^{\sec}\left(t\right))$ in Fig. 4a and c shows that there is no visible effect of the ZFS in the traces $V_{+1}^{\text{non-sec}}\left(t\right)$ and $V_{-1}^{\text{non-sec}}\left(t\right)$ , and they look virtually identical to $V_{+1}^{\sec}\left(t\right)$ and $V_{-1}^{\sec}\left(t\right)$ . The frequency shift $\delta_{\mathrm{ZFS}}\mathrm{sin}\left(2\beta_{\mathrm{dip}}\right)\omega_{\mathrm{dip}}$ seems to be averaged out after integration for these terms. The situation is different in the case of $V_{0}^{\text{non-sec}}\left(t\right)$ and $V_{0}^{\sec}\left(t\right)$ in Fig. 4b. Whereas $V_{0}^{\sec}\left(t\right)$ is a constant function of time and does not contribute to the echo modulation, $V_{0}^{\text{non-sec}}(t)$ shows a continuous decay of the echo intensity with increasing time. This decay does not contain any additional dipolar oscillations, and its shape does not seem to follow any obvious simple mathematical law. For the full LaserIMD traces in Fig. 4d, this means that, whereas the trace $V_{\mathrm{LaserIMD}}^{\sec}(t)$ looks like a S=1/2 DEER trace with a modulation depth of $\lambda =66.\bar{6}\;\%$ when not considering the ZFS, the trace $V_{\mathrm{LaserIMD}}^{\text{non-sec}}\left(t\right)$ with the ZFS shows the same dipolar oscillations but on top of a decay. Moreover, this means that, due to the coherence transfer pathway with $\Delta m_{T}=0$ also resulting in a variation in the echo intensity, the modulation depth of LaserIMD is increased by the ZFS, and values higher than $66.\bar{6}\;\%$ can be reached.

**Figure 5:**
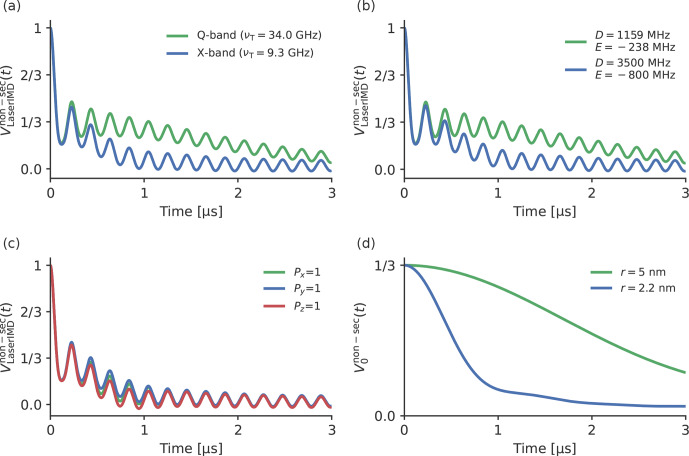
A comparison of different LaserIMD traces $V_{\mathrm{LaserIMD}}^{\text{non-sec}}(t)$ with different parameters. The following values were used for the simulations. In panel **(a)**, TPP, r=2.2 nm, and $\nu_{T}=34.0$ GHz (green) and $\nu_{T}=9.3$ GHz (blue). In panel **(b)**, $P_{x}=0.33$ , $P_{y}=0.41$ and $P_{z}=0.26$ ; r=2.2 nm; $\nu_{T}=9.3$ GHz; D=1159 and E=-238 MHz (green); and D=3500 and E=-800 MHz (blue). In panel **(c)**, D=1159 MHz and E=-238 MHz; r=2.2 nm; $\nu_{T}=9.3$ GHz; $P_{x}=1$ , $P_{y}=0$ and $P_{z}=0$ (green); $P_{x}=0$ , $P_{y}=1$ and $P_{z}=0$ (blue); and $P_{x}=0$ , $P_{y}=0$ and $P_{z}=1$ (red). In panel **(d)**, TPP, $\nu_{T}=34.0$ GHz, and r=2.2 nm (blue) and r=5.0 nm (green).

**Figure 6:**
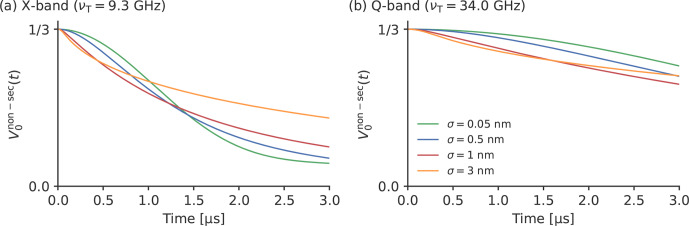
The influence of the width of the distance distribution on the decay of $V_{0}^{\text{non-sec}}\left(t\right)$ for TPP in the **(a)** X-band and **(b)** Q-band. The simulations were performed for a Gaussian distance distribution width a mean of 3.0 nm and different standard deviations σ .

The frequency shift caused by the non-secular terms of the ZFS in LaserIMD depends not only on D and E but also on the zero-field populations ( $P_{x}$ , $P_{y}$ and $P_{z}$ ), the Zeeman frequency $\nu_{T}$ and the distance r (see Eqs. 21–24). The influence of these parameters was studied by simulating additional LaserIMD traces with different magnetic field strengths, ZFS values, zero-field populations and distance distributions (see Figs. 5 and 6). In Fig. 5a, two LaserIMD traces in X- and Q-band ( $\nu_{T}=9.3$ and $\nu_{T}=34.0$ GHz) with TPP as a transient triplet label and a distance of r=2.2 nm are compared. Figure 5b shows the comparison between the ZFS of TPP ( D=1159 and E=-238 MHz) and a stronger ZFS of D=3500 and E =-800 MHz, as such high values are possible for some labels like rose bengal and erythrosin B (Williams et al., 2020; Bertran et al., 2022b). Both simulations were performed in Q-band with r=2.2 nm. Figure 5c shows three simulations with the population of the zero-field triplet states being completely assigned to $P_{x}$ , $P_{y}$ or $P_{z}$ . In Fig. 5d, the effect of different distances of r=2.2 and r=5.0 nm on $V_{0}^{\text{non-sec}}\left(t\right)$ is shown for TPP in Q-band. The simulations in Fig. 5 were all done with a single distance. To study the influence of the width of the distance distribution on $V_{0}^{\text{non-sec}}\left(t\right)$ , additional simulations were performed with a Gaussian distance distribution with a mean of 3.0 nm and different standard deviations σ ranging from 0.05 to 3.0 nm. The results of these simulations are shown in Fig. 6a and b for X-band and Q-band, respectively.

Figure 5a, b and c show that there are no visible differences in the dipolar oscillations in $V_{+1}^{\text{non-sec}}\left(t\right)$ and $V_{-1}^{\text{non-sec}}\left(t\right)$ when the Zeeman frequency, ZFS or zero-field populations are changed. This can also be seen in the Supplement S4, S5 and S6, where the traces for different Zeeman frequencies, ZFSs and distances are compared in more detail. This agrees with the former results in Fig. 4: the frequency shift due to the ZFS is virtually averaged out in a powder sample for $V_{+1}^{\text{non-sec}}\left(t\right)$ and $V_{-1}^{\text{non-sec}}\left(t\right)$ , so changing the involved parameters should also have little effect. The situation is different for $V_{0}^{\text{non-sec}}\left(t\right)$ , which, as is shown in Fig. 4c, is more strongly affected by the ZFS. The previously mentioned, decay is faster for lower Zeeman frequencies (see Fig. 5a) and a stronger ZFS (see Fig. 5b). Because $\delta_{\mathrm{ZFS}}$ ultimately depends on the ratio of the ZFS to the Zeeman frequency, a higher ZFS and a lower Zeeman frequency both increase the magnitude of the frequency shift in $\omega_{0}^{\text{non-sec}}$ in the same way, leading to the same effect on the LaserIMD trace. The parameters that have the least influence on the LaserIMD trace are the zero-field populations (see Fig. 5c). Changing the populations of the zero-field states does not seem to affect the dipolar oscillations, as was the case for different ZFSs and magnetic field strengths. This time, the decay of $V_{0}^{\text{non-sec}}\left(t\right)$ is also barely affected by different zero-field populations. Figure 5d shows that shorter distances lead to a faster decay of $V_{0}^{\text{non-sec}}\left(t\right)$ . As can be seen in Eqs. (21)–(23), changing the distance r from 2.2 to 5.0 nm leads to an increase in the LaserIMD frequencies $\omega_{+1}^{\text{non-sec}}$ , $\omega_{0}^{\text{non-sec}}$ and $\omega_{-1}^{\text{non-sec}}$ that scales with $r^{-3}$ . This distance dependence of the dipolar oscillations (not shown in Fig. 5c) is used in PDS for the calculation of the distance distributions. In the case of LaserIMD, the steepness of the decay of $V_{0}^{\text{non-sec}}\left(t\right)$ is an additional feature that depends on the distance between the spin labels. As can be seen in Fig. 6, the width of the distance distribution also has an influence on the decay of $V_{0}^{\text{non-sec}}\left(t\right)$ . In X-band (see Fig. 6a) and for small standard deviations of σ=0.05 nm, $V_{0}^{\text{non-sec}}\left(t\right)$ has a sigmoid-like shape. Increasing the width has a twofold effect on the decay of $V_{0}^{\text{non-sec}}\left(t\right)$ . Whereas the initial decay is steeper, on a long scale, the decay of $V_{0}^{\text{non-sec}}\left(t\right)$ is decreased for broader distance distributions. This can clearly be seen in the case of σ=3.0 nm: for t<1 µ s, $V_{0}^{\text{non-sec}}\left(t\right)$ decays faster for the simulation with σ=3.0 nm than with σ=0.05 nm; for t>1 µ s, $V_{0}^{\text{non-sec}}\left(t\right)$ decays slower for σ=3.0 nm than for σ=0.05 nm. In Q-band, where the decay of $V_{0}^{\text{non-sec}}\left(t\right)$ is generally slower, the simulations in Fig. 6b show that only the first effect is of relevance here. It can be seen that the first part of the decay of $V_{0}^{\text{non-sec}}\left(t\right)$ is again steeper for broader distance distributions, but the second part, where this behavior is inverted, lies outside the time window. This means that, in Q-band, the width of the distance distribution has a smaller influence on the decay of $V_{0}^{\text{non-sec}}\left(t\right)$ than in X-band.

Taken together, variations in the ZFS parameter, the population of the ZFS states and the employed magnetic field (X- or Q-band) do not affect the dipolar oscillations in $V_{+1}^{\text{non-sec}}\left(t\right)$ and $V_{-1}^{\text{non-sec}}\left(t\right)$ . They mostly have an effect on the decay of $V_{0}^{\text{non-sec}}\left(t\right)$ , such that larger ZFS parameters and lower magnetic fields will lead to a stronger additional decay in the LaserIMD trace. The additional decay also depends on the distance distribution between the spin labels: it is faster for shorter distances, and the shape of the decay also depends on the width of the distance distribution (in X-band more than in Q-band). The decay of $V_{0}^{\text{non-sec}}\left(t\right)$ can, therefore, be used as an additional source of information for the calculation of the distance distribution.

So far, all simulations only showed a visible effect of the ZFS on $V_{0}^{\text{non-sec}}\left(t\right)$ , but no significant influence on $V_{+1}^{\text{non-sec}}\left(t\right)$ and $V_{-1}^{\text{non-sec}}\left(t\right)$ was observed. To check if and when the ZFS also has an influence on $V_{+1}^{\text{non-sec}}\left(t\right)$ and $V_{-1}^{\text{non-sec}}\left(t\right)$ , we performed additional simulations where the effect of the ZFS is expected to be stronger. This can be obtained by either lower Zeeman frequencies or higher ZFS values. As the effect on $\delta_{\mathrm{ZFS}}$ is the same in both cases, the ratio of D and the Zeeman frequency of the triplet $\nu_{T}$ can be defined as follows:

38 $$q=\frac{D}{2\pi \nu_{T}}.$$

For simplification, the ZFS was assumed to be axial with E=0 . This simplifies the expression of $\delta_{\mathrm{ZFS}}$ to

39 $$\delta_{\mathrm{ZFS}}=\frac{3}{4}q\mathrm{sin}\left(2\beta_{T}\right)\mathrm{cos}\left(\alpha_{T}\right).$$

**Figure 7:**
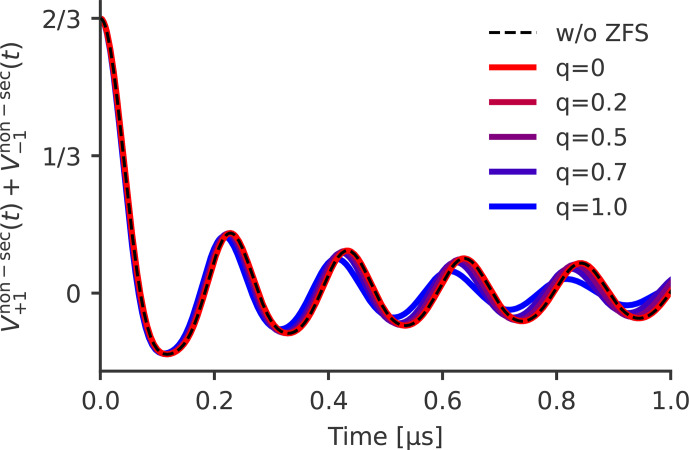
The sum of $V_{+1}^{\text{non-sec}}\left(t\right)$ and $V_{-1}^{\text{non-sec}}\left(t\right)$ for different values of q and $P_{x}=0.33$ , $P_{y}=0.41$ , $P_{z}=0.26$ and r=2.2 nm. Only the real part is shown.

The simulation in X-band with TPP from Fig. 4 corresponds to a ratio where q is approximately 0.13. Here, we tried values for q of up to 1. Figure 7 shows the sum of $V_{+1}^{\text{non-sec}}\left(t\right)$ and $V_{-1}^{\text{non-sec}}\left(t\right)$ of these simulations and compares it to a trace where the effect of the ZFS has been ignored. It can be seen that the traces are negligibly affected by the ZFS up to q=0.5 . For higher values, the dipolar oscillations start to get shifted to slightly higher frequencies and are also smoothed out more quickly. Analyzed with the oversimplified kernel $K_{\mathrm{DEER}}(t,r)$ of the S=1/2 model, this would result in a shift to smaller distances and an artificial broadening of the distance distribution. However, for experimentally relevant distance distributions with a finite width, the oscillations typically fade out much quicker, and cases where four oscillations can be resolved are scarce. In such a case, the observed influence of the ZFS for high values of q can be expected to be almost negligible. Furthermore, as q=1 is equivalent to a ZFS that is of the same order of magnitude as the Zeeman frequency, this is not relevant for most practical applications, as LaserIMD is typically performed in X- or Q-band ( $\nu_{T}=9.3$ or $\nu_{T}=34.0$ GHz), and all transient triplet labels used so far have a ZFS value D below 4 GHz (Dal Farra et al., 2019b; Williams et al., 2020). Even in the most extreme case, this would result in q values smaller than 0.5. Consequently, the effect of the ZFS on $V_{+1}^{\text{non-sec}}\left(t\right)$ and $V_{-1}^{\text{non-sec}}\left(t\right)$ is not relevant for most experiments and, even though the $V_{+1}^{\text{non-sec}}\left(t\right)$ and $V_{-1}^{\text{non-sec}}\left(t\right)$ can, in principle, be influenced by the ZFS, it seems to be a safe assumption that the ZFS in LaserIMD affects only the decay in $V_{0}^{\text{non-sec}}\left(t\right)$ and not the dipolar oscillations in $V_{+1}^{\text{non-sec}}\left(t\right)$ and $V_{-1}^{\text{non-sec}}\left(t\right)$ .

As previously stated, in the secular approximation, LaserIMD traces can be analyzed with the kernel $K_{\mathrm{DEER}}(t,r)$ of the S=1/2 model. To examine the extent to which this is true when the ZFS is not negligible, we simulated LaserIMD traces that were subsequently analyzed with $K_{\mathrm{DEER}}(t,r)$ . To mimic experimental conditions more closely, we assumed an incomplete excitation of the transient triplet label, and the intermolecular dipolar background was also considered. TPP was used as a transient triplet label with a distance to the permanent spin label of r=2.2 nm and a modulation depth of λ=50% , which roughly correspond to the values that can be typically achieved in experiments. Simulations were performed in X- and Q-band with different background decay rates varying between k=0.0 µ s ${}^{-1}$ (no background) and k=0.4 µ s ${}^{-1}$ . The resulting traces were then analyzed with $K_{\mathrm{DEER}}(t,r)$ and Tikhonov regularization (see Supplement S7 for details).

**Figure 8:**
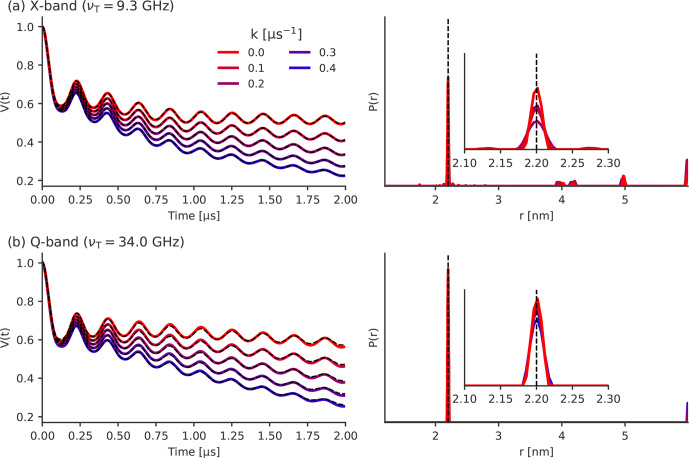
Simulated LaserIMD traces $V_{\mathrm{LaserIMD}}^{\text{non-sec}}\left(t\right)$ including the ZFS for TPP as a transient triplet label and r=2.2 nm in the **(a)** X-band ( $\nu_{T}=9.3$ GHz) and **(b)** Q-band ( $\nu_{T}=34.0$ GHz). The background decay that was used for the simulation was varied between k=0.0 and k=0.4 µ s ${}^{-1}$ . The left side shows the simulated traces (with the fits as a dashed black line), and the right side shows the distance distributions that were obtained with Tikhonov regularization with $K_{\mathrm{DEER}}(t,r)$ . The true distance of r=2.2nm is plotted as a dashed black line.

**Table 1:** The background decay values and modulation depths that were determined for the simulations from Fig. 8. The modulation depth for the simulations was always set to λ=50 %.

	X-band ( $\nu_{T}=9.3$ GHz)		Q-band ( $\nu_{T}=34.0$ GHz)	
k [ µ s ${}^{-1}$ ]	$k_{\mathrm{fit}}$ [ µ s ${}^{-1}$ ]	$\lambda_{\mathrm{fit}}$ [%]	$k_{\mathrm{fit}}$ [ µ s ${}^{-1}$ ]	$\lambda_{\mathrm{fit}}$ [%]
0.0	0.00	47	0.07	32
0.1	0.00	54	0.17	32
0.2	0.00	61	0.26	33
0.3	0.00	66	0.35	34
0.4	0.01	70	0.44	36

The simulations and fitted distance distributions can be seen in Fig. 8, and the background decay rates and modulations depths that were obtained by the fits are shown in Table 1. Figure 8 shows that the fits agree well with the simulated data, and the main peak of the distance distribution at r=2.2 nm is fitted appropriately in X- as well as in Q-band. However, there can be additional artifact peaks in the distance distributions, and the fitted modulation depths and background decay rates can be erroneous (see Table 1). This is particularly pronounced in X-band, which shows artifacts in the distance distribution between 3.9 and 5.0 nm and at the higher-distance end. Moreover, the background decay rates and modulation depths deviate significantly from the values that were originally used for the simulations. The simulations in X-band are always fitted with a background decay rate close to zero ( $k_{\mathrm{fit}}\approx 0.0$ µ ms ${}^{-1}$ ), even in the cases where the strongest background was included ( k=0.4 µ ms ${}^{-1}$ ) in the simulation. The modulation depth was fitted with values from 47 % to 70 % and varies significantly for different background decays. In Q-band, the fitted parameters are closer to the input values of the simulations. The distance artifacts that appeared in X-band between 3.9 and 5.0 nm have disappeared, and only those at the long distance limit remain. In Q-band, the fitted background decay is always a bit larger than the true value. Except for the case were the true background decay is set to k=0 µ s ${}^{-1}$ , the deviation of the fitted and the true background decay is smaller in Q-band than in X-band. Only the obtained modulation depths are less accurate than in X-band and fitted to values between 32 % and 36 %. Although these simulations are only anecdotal evidence and generalizations from these data must be taken with caution, they show that it is possible to extract the main distance peak correctly when LaserIMD data are analyzed with $K_{\mathrm{DEER}}(t,r)$ . Thus, analyzing LaserIMD traces with $K_{\mathrm{DEER}}(t,r)$ can be an option in situations where the ZFS values and zero-field populations of the transient triplet label are unknown and their effect cannot be included in the analysis. However, this way of analyzing LaserIMD data can give artifacts at higher distances as well as errors in the obtained modulation depth and background decay rate. This is particularly pronounced for low magnetic fields (e.g., X-band), and similar results can be expected for transient triplet labels with higher ZFS values.

### 4.2 LiDEER simulations

In LaserIMD, transient triplet labels of all orientations are excited by the laser flash and contribute to the signal; thus, an integration over all orientations was performed (Eqs. 25–27) to calculate the LaserIMD signal. In contrast, the transient triplet labels are additionally excited by microwave observer pulses in LiDEER. As the spectrum of many transient triplet labels exceeds the excitation bandwidth of these pulses (Di Valentin et al., 2014; Williams et al., 2020; Krumkacheva et al., 2019), only a small number of orientations within the excitation bandwidth contribute to the signal. Because the frequency shift $\delta_{\mathrm{ZFS}}$ of the LiDEER frequencies (Eqs. 34–37) depends on the orientation of the transient triplet labels, the choice of the observer frequency influences the shape of the LiDEER trace.

**Figure 9:**
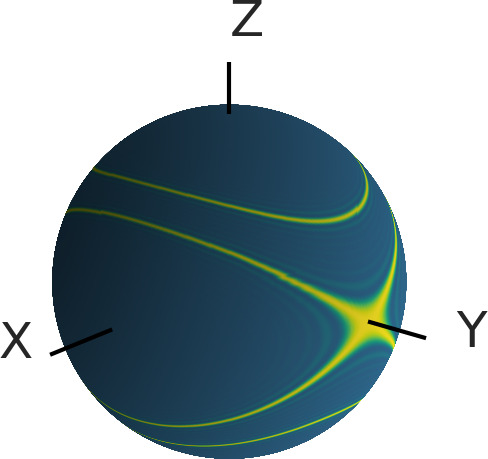
The orientations (shown in yellow) of the transient triplet label that are excited by a rectangular π pulse with a pulse length of 20 ns that is placed on the $Y^{+}$ peak of EPR spectrum of TPP in Q-band. For the calculation, the magnetic field was set to B=1.2097T , and the pulse frequency was set to 33.646 GHz. The position of the pulse relative to the EPR spectrum is shown in Fig. S7. The angle $\beta_{T}$ is the polar angle of the depicted sphere, and the angle $\gamma_{T}$ is the azimuthal angle.

In experiments in which the commonly used nitroxides or other spin labels with $g_{D}\approx 2$ are used as pump spin, the resonator bandwidth allows one to use only the $Y^{\pm }$ peaks as the observer position, as the other parts of the EPR spectrum of the transient triplet label lie outside the resonator bandwidth (Bieber et al., 2018; Bowen et al., 2021). Figure 9 shows the orientations of the triplet label TPP that, in this case, contribute to the LiDEER signal. The contribution of the orientations where the Y axis of eigenframe of the ZFS is parallel to the external magnetic field ( $\beta_{T}=\pi /2$ and $\gamma_{T}=\pi /2)$ is eponymous for the $Y^{\pm }$ peaks. For this orientation, the frequency shift $\delta_{\mathrm{ZFS}}=0$ , and the ZFS has no effect on the LiDEER trace. However, it can be seen that other orientations are also excited if the observer pulses are placed on either of the $Y^{\pm }$ peaks. For these contributions, it cannot guaranteed that $\delta_{\mathrm{ZFS}}$ is always zero, so that there might still be an effect of the ZFS.

To study the effect of the ZFS in LiDEER, numerical time-domain simulations for different ZFS values in X- and Q-band were performed. The microwave pulses were placed on the $Y^{+}$ peak of the EPR spectrum and had a finite length, power and bandwidth so that only the orientations that are shown in Fig. 9 contribute to the LiDEER signal, as is the case in the experimental setup. A simulation for TPP as a transient triplet label was performed in X- and Q-band, and an additional simulation with a larger ZFS of D=3500 and E=-800 MHz was performed in X-band. The permanent spin label was included as a doublet spin with an isotropic g value ( $g_{D}=2)$ and without any additional hyperfine interactions. The distance was set to r=2.2 nm, and no background from intermolecular spins was included. To check for artifacts that occur in distance distributions if the ZFS is ignored in data analysis, the simulated LiDEER traces were analyzed with $K_{\mathrm{DEER}}(t,r)$ and Tikhonov regularization. The details of the calculation of the distance distribution are given in Supplement S7, and the details of the simulations can be found in Supplement S8.

**Figure 10:**
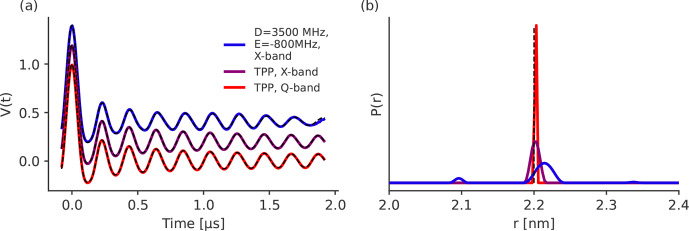
LiDEER simulations with the observer pulse placed on the $Y^{+}$ -peak of the EPR spectrum of the transient triplet label in different frequency bands and with different ZFS. The traces are shifted by 0.2 for better visibility. For the simulation in Q-band and the parameters of TPP, the magnetic field was set to 1.2097 T and the observer frequency was set to 33.64 GHz. For the simulations in X-band, the magnetic field was set to 0.33 T. For the X-band simulation with the ZFS values of TPP, the observer frequency was set to 9.042 GHz, and for the simulation with ZFS values of D=3500 and E=-800 MHz, the observer frequency was set to 9.042 GHz. The position of the observer and pump pulse with respect to the EPR spectrum is shown in Fig. S7a, c and e. The further parameters were $P_{x}=0.33$ , $P_{y}=0.41$ , $P_{z}=0.26$ and r=2.2 nm. The numerical simulations were fitted with Tikhonov regularization. The fits are shown as dashed black lines. Panel **(b)** displays the corresponding distance distribution. The true distance of r=2.2 nm is plotted as a dashed black line.

Figure 10a shows the simulated LiDEER traces, and Fig. 10b presents the obtained distance distributions. The differences in the LiDEER traces for different ZFS and Zeeman frequencies are smaller than they are in LaserIMD (see Fig. 4). This is because, in LiDEER, there is no equivalence for the coherence transfer pathway with $\Delta m_{T}=0$ that showed the strongest dependency on the ZFS and magnetic fields in LaserIMD (see Fig. 5). The distance distribution for TPP in Q-band shows a narrow peak at 2.20 nm with a full width at half maximum ( FWHM) of 0.004 nm. This fits to the 2.20 nm ( FWHM=0 nm) that was used for the simulation. In X-band, the distance distribution with TPP is also centered at 2.20 nm but gets broadened to a FWHM of 0.014 nm. This trend increases for the large ZFS with D=3500 and E=-800 MHz in X-band. Here, the distance distribution gets even broader with an FWHM of 0.028 nm and is now also shifted to a center of ≈2.22 nm. This behavior fits with the results of LaserIMD in Fig. 7, where the shifts in the dipolar oscillation also get larger when the ZFS is large compared with the Zeeman frequency. However, it must also be stated that the observed shifts in the distance distribution are still rather small here and should be below the resolution limit that is relevant in most experiments. Additional traces in which the observer pulse was set off-resonance to the canonical peaks were also performed and are presented in Supplement S9. Here, the effect of the ZFS can clearly be seen, and the LiDEER trace of the simulation with D=3500 and E=-800 MHz in X-band shows strong deviations from the other traces that were simulated with a smaller ZFS. The dipolar oscillations fade out much faster, which also leads to a stronger broadening of the distance distributions. However, for experimentally relevant cases with distance distributions of a finite width, the oscillations in the dipolar trace fade out much faster anyway. It is to be expected that, in these cases, the effect of the ZFS on the LiDEER trace are rather small and that artifacts in the distance distribution are, therefore, not so pronounced, even in the case when the observer pulses are set to a non-canonical orientation.

This means that, in general, the ZFS has an effect on LiDEER and the LiDEER trace changes when different parts of the EPR spectrum of the transient triplet label are used for excitation by the observer pulses. However, in the special case when either of the $Y^{\pm }$ peaks is used as the position for the observer pulse, the effect of the ZFS can be suppressed and LiDEER traces can be analyzed with the $K_{\mathrm{DEER}}(t,r)$ kernel without introducing significant artifacts in the distance distribution. This is particularly valid for TPP – and other transient triplet labels with a similar ZFS – in Q-band.

### 4.3 Experiments

To experimentally confirm the theoretical finding that the ZFS has an influence on the shape of the LaserIMD trace, LaserIMD measurements were performed at different magnetic field strengths in X- and Q-band and with two model systems with shorter and longer distances between the labels. This should result in scenarios were the ZFS has either a weak effect on the trace (high magnetic field strength and long distance) or a strong effect on the trace (low magnetic field strength and short distance). The LaserIMD experiments were simulated with the newly derived model that includes the ZFS. The distance distributions and background decay rates that were used for these simulations of the LaserIMD traces were determined with LiDEER. The measurements were performed with the peptides TPP–pAA ${}_{5}$ –NO ⚫ and TPP–pAA ${}_{10}$ –NO ⚫ . They contain TPP as a transient triplet label and the nitroxide 2,2,6,6-tetramethylpiperidine-1-oxyl-4-amino-4-carboxylic acid (TOAC) as permanent spin label. Both labels are separated by a rather rigid helix consisting of l-alanine and α -isobutyric acid (Di Valentin et al., 2016).

**Figure 11:**
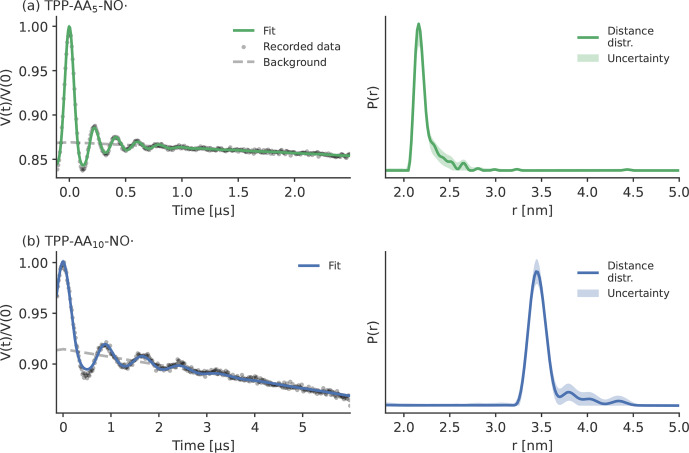
Experimental LiDEER data of the two peptides, all recorded in Q-band at 30 K in MeOD / D ${}_{2}$ O ( 98/2 vol %). Panel **(a)** shows TPP–pAA ${}_{5}$ –NO ⚫ , and panel **(b)** displays TPP–pAA ${}_{10}$ –NO ⚫ . The raw data are depicted on the left side as gray dots with the fits as a straight line, and the background fit is depicted as a dashed gray line. The distance distributions obtained with Tikhonov regularization (Fábregas Ibáñez et al., 2020) are shown on the right side. The shaded areas correspond to the 95 % confidence intervals that were obtained with bootstrapping.

So far, the LaserIMD simulations that were described above mostly only invoked a single delta-like distance. To simulate LaserIMD for an entire distance distribution in a fast way, the dipolar kernel $K_{\mathrm{LaserIMD}}^{\text{non-sec}}\left(t,r\right)$ needs to be calculated. Therefore, we implemented a C ++ software tool that can perform the numerical integration of Eqs. (25)–(27) to calculate $S_{\mathrm{LaserIMD}}^{\text{non-sec}}\left(t,r\right)$ . It allows the user to specify different ZFS values, zero-field populations and Zeeman frequencies. The background decay and modulation depth can then be included afterwards to obtain the full kernel $K_{\mathrm{LaserIMD}}^{\text{non-sec}}\left(t,r\right)$ (see Eq. 29). The obtained kernel can, for example, be used in combination with the DeerLab software (Fábregas Ibáñez et al., 2020) to analyze experimental LaserIMD traces. The program, including its source code, is available at GitHub (https://github.com/andreas-scherer/LaserIMD_kernel, last access: 21 December 2022). Here, it was used to calculate the kernel that corresponds to the experimentally determined parameters for TPP of the peptides TPP–pAA ${}_{5}$ –NO ⚫ and TPP–pAA ${}_{10}$ –NO ⚫ (ZFS values of D=1159 and E=-238 MHz and zero-field populations of $P_{x}=0.33$ , $P_{y}=0.41$ and $P_{z}=0.26$ ; Di Valentin et al., 2014) at the Zeeman frequencies that correspond to the used magnetic field strengths ( $\nu_{T}=9.28$ and $\nu_{T}=9.31$ GHz in X-band and $\nu_{T}=34.00$ GHz in Q-band; see also Supplement S10). The distance distributions of TPP–pAA ${}_{5}$ –NO ⚫ and TPP–pAA ${}_{10}$ –NO ⚫ that were used for the LaserIMD simulations were obtained by LiDEER measurements. LiDEER traces were recorded in Q-band with the observer pulse placed on the $Y^{-}$ peak and analyzed with $K_{\mathrm{DEER}}(t,r)$ and Tikhonov regularization, as the simulations in Sect. 4.2 showed that no artifacts are to be expected in this case. More details on the experiments and distance calculations can be found in Supplement S7 and S10. The results of the LiDEER measurements are shown in Fig. 11, and the extracted distance distributions exhibit a narrow peak at 2.2 nm for TPP–pAA ${}_{5}$ –NO ⚫ and at 3.5 nm for TPP–pAA ${}_{10}$ –NO ⚫ , as expected (Bieber et al., 2018; Di Valentin et al., 2016). As the LaserIMD and LiDEER measurements have different modulation depths, the modulation depth of LiDEER ( $\lambda_{\mathrm{LiDEER}}$ ) cannot be used for the simulation of the LaserIMD. This makes the modulation depth of the LaserIMD traces ( $\lambda_{\mathrm{LaserIMD}}$ ) the only parameter that is missing for the simulations. Therefore, the simulated LaserIMD traces were fitted to the measured ones by rescaling the modulation depth. As the background decay rate depends linearly on the modulation depth (Hu and Hartmann, 1974; Pannier et al., 2000), it must be rescaled together with the modulation depth. For LaserIMD, we assume that coherence transfer pathways with $\Delta m_{T}=0$ do not contribute to the background, as the decay of the echo intensity is on a much longer timescale than the dipolar oscillations that constitute the main contribution of the intermolecular background. Therefore, we additionally reduce the rescaled background decay rate by a factor of 2/3 :

40KLaserIMDnon-sect,rλLaserIMD=exp⁡-23λLaserIMDλLiDEERkLiDEERt(λLaserIMDSLaserIMDnon-sect,r+1-λLaserIMD),41VLaserIMDtλLaserIMD=KLaserIMDnon-sect,rλLaserIMDPLiDEER(r).

The simulated LaserIMD trace $V_{\mathrm{LaserIMD}}{\left(t\right)}_{\lambda_{\mathrm{LaserIMD}}}$ was fitted to the experimental LaserIMD data by varying the modulation depth $\lambda_{\mathrm{LaserIMD}}$ so that the root-mean-square displacement of the simulated and experimental traces was minimized. Simulations without the effect of the ZFS were also performed in order to clearly see the difference between them and the simulations with the ZFS. For the simulations without the ZFS, the modulation depth of the LaserIMD simulations with the ZFS was taken because it was determined by the fit and reduced by a factor of 2/3 , as the coherence transfer pathway with $\Delta m_{T}=0$ no longer contributes to the echo modulation.

**Figure 12:**
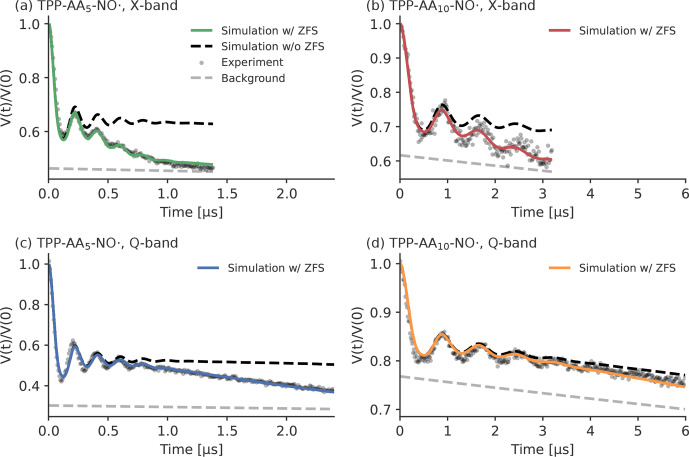
Experimental LaserIMD traces of the peptides, recorded at 30 K in MeOD / D ${}_{2}$ O ( 98/2 vol %). Panel **(a)** shows TPP–AA ${}_{5}$ –NO ⚫ in X-band ( $\nu_{T}=9.28$ GHz) (green), panel **(b)** shows TPP–AA ${}_{10}$ –NO ⚫ in X-band ( $\nu_{T}=9.31$ GHz) (red), panel **(c)** shows TPP–AA ${}_{5}$ –NO ⚫ in Q-band ( $\nu_{T}=34.00$ GHz) (blue) and panel **(d)** shows TPP–AA ${}_{10}$ –NO ⚫ in Q-band ( $\nu_{T}=34.00$ GHz) (orange). The colored traces show simulations that include the ZFS. The simulations without the effects of the ZFS are shown as a black dashed line. The experimentally recorded data are depicted as gray dots. The backgrounds of the simulations are shown as a gray dashed line. The simulations were performed with the distance distributions and background decays that were obtained by the LiDEER measurements.

The results of the LaserIMD measurements and the corresponding simulations are shown in Fig. 12. It can be clearly seen that the shape of the experimental traces changes depends on whether they were recorded in X- or Q-band: in X-band, the traces have a stronger decay than in Q-band. This is a first strong indication of the effect of the ZFS, as predicted by the simulations (see Fig. 5). The influence of the ZFS shows itself clearly in the differences between the experimental data and the simulations where the effect of the ZFS was ignored. In particular, the experimental LaserIMD traces show a stronger decay than the background decay of simulations without the ZFS. This difference is more pronounced in TPP–AA ${}_{5}$ –NO ⚫ than in TPP–AA ${}_{10}$ –NO ⚫ and also stronger in X-band than in Q-band. Thus, for TPP–AA ${}_{5}$ –NO ⚫ in X-band, the deviation between the simulations without the ZFS and the experiments is the largest, whereas it is nearly absent in the case of TPP–AA ${}_{10}$ –NO ⚫ in Q-band. This additional decay of the experimental traces cannot be explained without considering the effect of the ZFS, but it is understandable with a model that includes the ZFS. The stronger decay of the experimental traces can be assigned to the coherence transfer pathway with $\Delta m_{T}=0$ , which leads to an additional contribution to the LaserIMD trace $V_{0}^{\text{non-sec}}\left(t\right)$ with a continuously decaying signal (see Fig. 4). As shorter distances and lower magnetic fields lead to a stronger decay of $V_{0}^{\text{non-sec}}\left(t\right)$ , this also explains why the additional decay in the experimental data is stronger for TPP–AA ${}_{5}$ –NO ⚫ than for TPP–AA ${}_{10}$ –NO ⚫ and stronger in X-band than in Q-band. It is noteworthy that the model with the ZFS provides not only a qualitative but also a quantitative agreement between the experimentally recorded LaserIMD traces and the corresponding simulations.

To see how the additional decay of the ZFS affects the analysis of experimental LaserIMD traces, the recorded data were analyzed with Tikhonov regularization; the results that are obtained with a LaserIMD kernel that includes the ZFS are compared to those obtained by a DEER kernel that ignores the ZFS (see Supplement S11 for a detailed overview of the results). The comparison of the obtained distance distributions shows that, even when the ZFS is ignored, the main distance peak is obtained correctly in all cases. For the measurements in Q-band, the entire distance distributions turn out to be virtually identical, regardless of whether the ZFS is included in the analysis routine or not (see Fig. S13c–d). The situation is different in X-band. For TPP–AA ${}_{5}$ –NO ⚫ in X-band, the strong additional decay is interpreted as an additional artifact peak at around 5.0 nm if the ZFS is ignored (see Fig. S13a). This peak disappears when the ZFS is considered. For TPP–AA ${}_{10}$ –NO ⚫ in X-band, the analysis that ignores the ZFS also shows an additional peak around 7.0 nm. However, this artifact is not as pronounced as the one of TPP–AA ${}_{5}$ –NO ⚫ and disappears in the validation. For the modulation depths and the background decay rates, there are notable differences when the ZFS is considered or omitted (see Tables S5 and S6 in Supplement S11). In all cases, ignoring the ZFS leads to a reduced modulation depth. In Q-band, the modulation depth is reduced by a factor of ≈2/3 , meaning that the additional decay is completely assigned to the intermolecular background. In accordance with that, the background decay rates are larger when the ZFS is ignored. In X-band, these effects are not as pronounced. As the additional decay is partially fitted by introducing distance artifacts when ignoring the ZFS, the modulation depth is only reduced by a factor of 0.72 for TPP–AA ${}_{10}$ –NO ⚫ and by a factor of 0.84 for TPP–AA ${}_{5}$ –NO ⚫ .

These results show that ignoring the ZFS for the analysis of LaserIMD leads to artifacts in the obtained results. For TPP as transient spin label, the artifacts are not as prominent in Q-band. There, the additional decay mostly leads to a stronger background decay and reduced modulation depth, and the distance distribution remains virtually unchanged. In X-band, however, artifact peaks in the distance distribution can occur if the ZFS is ignored.

## 5 Conclusion and outlook

In light-induced PDS, the ZFS interaction of the transient triplet label is a crucial parameter that can alter the shape of the dipolar traces. This implies that, in contrast to the former assumption, the spin system in LaserIMD and LiDEER cannot be treated in the secular approximation where the spin system behaves as if it would consist of two S=1/2 spins. A theoretical description of LaserIMD and LiDEER that also includes non-secular terms was developed, and it was shown that the dipolar frequencies depend on the magnitude of the ZFS and the Zeeman frequency (i.e., the external magnetic field). Time-domain simulations showed that, in LiDEER, this effect of the ZFS can be suppressed by exciting either of the $Y^{\pm }$ peaks with the observer pulses and by using transient triplet labels whose ZFS is small compared with the Zeeman frequency, such as TPP in Q-band. For experimental LiDEER data that are recorded under such conditions, the effect of the ZFS is negligible and a standard DEER kernel that does not consider the ZFS can be employed for data analysis.

In LaserIMD, simulations and experiments confirmed that there is an influence of the ZFS on the dipolar trace. It virtually does not affect the dipolar oscillation of the coherence transfer pathways with $\Delta m_{T}=\pm 1$ , but it is manifested in an additional decay of the LaserIMD trace. This decay is caused by the third coherence transfer pathway with $\Delta m_{T}=0$ , which was formerly believed not to contribute to the signal. The strength of this additional decay primarily depends on the ratio of the ZFS to the Zeeman frequency as well as on the distance between the transient and permanent spin label: it is stronger for a larger ZFS, lower magnetic fields and shorter distances. A software tool for the calculation of LaserIMD kernels that considers the influence of the ZFS was developed. It is available at GitHub (https://github.com/andreas-scherer/LaserIMD_kernel) and allows one to specify different ZFS values, zero-field populations and Zeeman frequencies. The feasibility of the new kernel was proven by experimentally recorded LaserIMD traces. A DEER kernel that ignores the ZFS cannot fit these traces correctly, and strong derivations between the experimental data and simulations can be observed. However, with the newly developed model that considers the ZFS, excellent fits of the experimental data were produced. The analysis of the experimental and simulated LaserIMD data with Tikhonov regularization showed that ignoring the ZFS compromises the obtained results. For transient triplet labels with a ZFS of ≈1 GHz, like TPP, this is no that problematic in Q-band. There, only the obtained modulation depths and background decay rates are affected if the ZFS is ignored; the distance distribution remains unchanged. In X-band, however, ignoring the ZFS is more severe and can additionally lead to artifact peaks in the distance distributions. This shows that the ZFS can have a significant impact in LaserIMD and should be considered when experimental data are analyzed.

## Supplement

10.5194/mr-4-27-2023-supplement The supplement related to this article is available online at: https://doi.org/10.5194/mr-4-27-2023-supplement.

## Data Availability

The raw data can be downloaded from https://doi.org/10.5281/zenodo.7283499 (Scherer et al., 2022b).

## Code availability

The source code for the LaserIMD kernel can be downloaded from https://doi.org/10.5281/zenodo.7576913 (Scherer et al., 2023a; https://github.com/andreas-scherer/LaserIMD_kernel). The source code for the time-domain LiDEER simulations can be downloaded from https://doi.org/10.5281/zenodo.7580933 (Scherer et al., 2023b; https://github.com/andreas-scherer/LiDEER_simulations.git).
