# Supplementary material for: The effect of the zero-field splitting in light-induced pulsed dipolar electron paramagnetic resonance (EPR) spectroscopy
Source: Magn Reson (Gott). 2023 Feb 8;4(1):27–46. doi: 10.5194/mr-4-27-2023 (PMC10583298; doi:10.5194/mr-4-27-2023)
Supplement: The supplement related to this article is available online at: https://doi.org/10.5194/mr-4-27-2023-supplement. [file mr-4-27-supplement.pdf]

Supplement of Magn. Reson., 4, 27–46, 2023
https://doi.org/10.5194/mr-4-27-2023-supplement
© Author(s) 2023. CC BY 4.0 License.

Supplement of

The effect of the zero-field splitting in light-induced pulsed dipolar electron paramagnetic resonance (EPR) spectroscopy

Andreas Scherer et al.

Correspondence to: Malte Drescher (malte.drescher@uni-konstanz.de)

The copyright of individual parts of the supplement might differ from the article licence.

Table of contents

S1 Theoretical derivation 3

S2 Influence of the remaining non-secular terms 6

S3 Convergence of the integral 7

S4 LaserIMD simulations for different Zeeman frequencies 9

S5 LaserIMD simulations for different ZFS values 10

S6 LaserIMD simulations for different distances 11

S7 Calculation of distance distributions with Tikhonov regularization 12

S8 LiDEER simulations 13

S9 Results of the LiDEER simulations on a non-canonical orientation 16

S10 EPR experiments 17

S11 Analysis of the experimental LaserIMD data 21

S12 References 27

S1 Theoretical derivation

The Hamiltonian is a sum of the secular and non-secular parts:

$$\hat{H} = \hat{H}^{\mathrm{sec}} + \hat{H}^{\mathrm{non-sec}}, \tag{S1}$$

With

$$\hat{H}^{\mathrm{sec}} = 2\pi\nu_{\mathrm{D}}\hat{S}_{\mathrm{D},z} + 2\pi\nu_{\mathrm{T}}\hat{S}_{\mathrm{T},z} + \left(\frac{D(3\cos^2(\beta_{\mathrm{T}})-1)}{6} + \frac{E\sin^2(\beta_{\mathrm{T}})\cos(2\gamma_T)}{2}\right)\left(2\hat{S}_{\mathrm{T},z}\hat{S}_{\mathrm{T},z} - \left(\hat{S}_{\mathrm{T},x}\hat{S}_{\mathrm{T},x} + \hat{S}_{\mathrm{T},y}\hat{S}_{\mathrm{T},y}\right)\right) + \omega_{\mathrm{dip}}(3\cos^2\beta_{\mathrm{dip}} - 1)\hat{S}_{\mathrm{D},z}\hat{S}_{\mathrm{T},z} \tag{S2}$$

$$\begin{aligned}\hat{H}^{\mathrm{non-sec}} = &\left(\frac{D\sin 2\beta_{\mathrm{T}}\exp(-i\alpha_{\mathrm{T}})}{4} - \frac{E\sin(\beta_{\mathrm{T}})\left(\cos(\beta_{\mathrm{T}})\cos(2\gamma_{\mathrm{T}}) - i\sin(2\gamma_{\mathrm{T}})\right)\exp(-i\alpha_{\mathrm{T}})}{2}\right)\left(\hat{S}_{\mathrm{T},z}\hat{S}_{\mathrm{T},+} + \hat{S}_{\mathrm{T},+}\hat{S}_{\mathrm{T},z}\right) \\ &+ \left(\frac{D\sin 2\beta_{\mathrm{T}}\exp(i\alpha_{\mathrm{T}})}{4} - \frac{E\sin(\beta_{\mathrm{T}})\left(\cos(\beta_{\mathrm{T}})\cos(2\gamma_{\mathrm{T}}) + i\sin(2\gamma_{\mathrm{T}})\right)\exp(i\alpha_{\mathrm{T}})}{2}\right)\left(\hat{S}_{\mathrm{T},-}\hat{S}_{\mathrm{T},z} + \hat{S}_{\mathrm{T},-}\hat{S}_{\mathrm{T},z}\right) \\ &+ \frac{3\omega_{\mathrm{dip}}\sin 2\beta_{\mathrm{dip}}}{4}\hat{S}_{\mathrm{D},z}\hat{S}_{\mathrm{T},+} + \frac{3\omega_{\mathrm{dip}}\sin 2\beta_{\mathrm{dip}}}{4}\hat{S}_{\mathrm{D},z}\hat{S}_{\mathrm{T},-}\end{aligned} \tag{S3}$$

The secular Hamiltonian is already diagonal with the eigenvalues $E^{\mathrm{sec}}_{m_{\mathrm{D}},\, m_{\mathrm{T}}}$ (Abragam and Bleaney, 2012; Blank and Levanon, 2005):

$$E^{\mathrm{sec}}_{+\frac{1}{2},+1} = \frac{\omega_{\mathrm{D}}}{2} + \omega_{\mathrm{T}} + E^{\mathrm{sec}}_{\mathrm{ZFS}} + E^{\mathrm{sec}}_{\mathrm{dip}}, \tag{S4}$$

$$E^{\mathrm{sec}}_{+\frac{1}{2},\,0} = \frac{\omega_{\mathrm{D}}}{2} - 2E^{\mathrm{sec}}_{\mathrm{ZFS}}, \tag{S5}$$

$$E^{\mathrm{sec}}_{+\frac{1}{2},\,-1} = \frac{\omega_{\mathrm{D}}}{2} - \omega_{\mathrm{T}} + E^{\mathrm{sec}}_{\mathrm{ZFS}} - E^{\mathrm{sec}}_{\mathrm{dip}}, \tag{S6}$$

$$E^{\mathrm{sec}}_{-\frac{1}{2},+1} = -\frac{\omega_{\mathrm{D}}}{2} + \omega_{\mathrm{T}} + E^{\mathrm{sec}}_{\mathrm{ZFS}} - E^{\mathrm{sec}}_{\mathrm{dip}}, \tag{S7}$$

$$E^{\mathrm{sec}}_{-\frac{1}{2},0} = -\frac{\omega_{\mathrm{D}}}{2} - 2E^{\mathrm{sec}}_{\mathrm{ZFS}}, \tag{S8}$$

$$E^{\mathrm{sec}}_{-\frac{1}{2},-1} = -\frac{\omega_{\mathrm{D}}}{2} - \omega_{\mathrm{T}} + E^{\mathrm{sec}}_{\mathrm{ZFS}} + E^{\mathrm{sec}}_{\mathrm{dip}}. \tag{S9}$$

Here, the following abbreviations were used:

$$E^{\mathrm{sec}}_{\mathrm{ZFS}} = \frac{3\cos(\beta_{\mathrm{T}})^2 - 1}{6}D + \frac{\cos(2\gamma_T)\sin(\beta_{\mathrm{T}})^2}{2}E \tag{S10}$$

and

$$E^{\mathrm{sec}}_{\mathrm{dip}} = \frac{3\cos\left(\beta_{\mathrm{dip}}\right)^2 - 1}{2}\omega_{\mathrm{dip}}. \tag{S11}$$

The non-secular terms contribute the following off-diagonal elements to the Hamiltonian:

$$\left\langle +\frac{1}{2}, +1\middle|\hat{H}^{\mathrm{non-sec}}\middle| +\frac{1}{2}, 0\right\rangle = H^{\mathrm{non-sec}}_{\mathrm{ZFS}} + H^{\mathrm{non-sec}}_{\mathrm{dip}}, \tag{S12}$$

$$\left\langle +\frac{1}{2}, 0\middle|\hat{H}^{\mathrm{non-sec}}\middle| +\frac{1}{2}, -1\right\rangle = -H^{\mathrm{non-sec}}_{\mathrm{ZFS}} + H^{\mathrm{non-sec}}_{\mathrm{dip}}, \tag{S13}$$

$$\left\langle -\frac{1}{2}, +1\middle|\hat{H}^{\mathrm{non-sec}}\middle| -\frac{1}{2}, 0\right\rangle = H^{\mathrm{non-sec}}_{\mathrm{ZFS}} - H^{\mathrm{non-sec}}_{\mathrm{dip}}, \tag{S14}$$

$$\langle -\tfrac{1}{2}, 0 \big| \hat{H}^{\text{non-sec}} \big| -\tfrac{1}{2}, -1 \rangle = -H_{\text{ZFS}}^{\text{non-sec}} - H_{\text{dip}}^{\text{non-sec}}, \tag{S15}$$

With the following abbreviations

$$E_{\text{ZFS}}^{\text{non-sec}} = \frac{\sqrt{2}\sin(2\beta_{\text{T}})\exp(-i\alpha_{\text{T}})}{4} D - \frac{\sin(\beta_T)(\cos(\beta_{\text{T}})\cos(2\gamma_{\text{T}}) - i\sin(2\gamma_{\text{T}}))\exp(-i\alpha_{\text{T}})}{\sqrt{2}} E \tag{S16}$$

and

$$E_{\text{dip}}^{\text{non-sec}} = \frac{3\sqrt{2}\sin(2\beta_{\text{dip}})}{8} \omega_{\text{dip}}. \tag{S17}$$

Written in matrix-form, the full Hamiltonian is:

$$\hat{H} = \hat{H}^{\text{sec}} + \hat{H}^{\text{non-sec}} = \begin{pmatrix} \hat{A} & 0 \\ 0 & \hat{B} \end{pmatrix} \tag{S18}$$

$$\hat{A} = \begin{pmatrix} E_{+\frac{1}{2},+1}^{\text{sec}} & E_{\text{ZFS}}^{\text{non-sec}} + E_{\text{dip}}^{\text{non-sec}} & 0 \\ \bar{E}_{\text{ZFS}}^{\text{non-sec}} + E_{\text{dip}}^{\text{non-sec}} & E_{+\frac{1}{2},0}^{\text{sec}} & -E_{\text{ZFS}}^{\text{non-sec}} + E_{\text{dip}}^{\text{non-sec}} \\ 0 & -\bar{E}_{\text{ZFS}}^{\text{non-sec}} + \bar{E}_{\text{dip}}^{\text{non-sec}} & E_{+\frac{1}{2},-1}^{\text{sec}} \end{pmatrix}, \tag{S19}$$

$$\hat{B} = \begin{pmatrix} E_{-\frac{1}{2},+1}^{\text{sec}} & E_{\text{ZFS}}^{\text{non-sec}} - E_{\text{dip}}^{\text{non-sec}} & 0 \\ \bar{E}_{\text{ZFS}}^{\text{non-sec}} - \bar{E}_{\text{dip}}^{\text{non-sec}} & E_{-\frac{1}{2},0}^{\text{sec}} & -E_{\text{ZFS}}^{\text{non-sec}} - E_{\text{dip}}^{\text{non-sec}} \\ 0 & -\bar{E}_{\text{ZFS}}^{\text{non-sec}} - \bar{E}_{\text{dip}}^{\text{non-sec}} & E_{-\frac{1}{2},-1}^{\text{sec}} \end{pmatrix}. \tag{S20}$$

To calculate the eigenvalues of this Hamiltonian perturbation theory was used. $\hat{H}^{\text{non-sec}}$ only includes off-diagonal elements, so the first order corrections are zero. The second order corrections were calculated according to:

$$E_{m_{\text{D}}, m_{\text{T}}}^{\text{non-sec}} = E_{m_{\text{D}}, m_{\text{T}}}^{\text{sec}} + \sum_{m_{\text{D}}, m_{\text{T}} \neq m'_{\text{D}}, m'_{\text{T}}} \frac{\left| \langle m_{\text{D}}, m_{\text{T}} \big| \hat{H}^{\text{non-sec}} \big| m'_{\text{D}}, m'_{\text{T}} \rangle \right|^2}{E_{m_{\text{D}}, m_{\text{T}}}^{\text{sec}} - E_{m'_{\text{D}}, m'_{\text{T}}}^{\text{sec}}} \tag{S21}$$

For further simplification, energy difference of two adjacent levels of the triplet submanifolds were simplified to only include the Zeeman splitting:

$$E_{\pm\frac{1}{2}, +1}^{\text{sec}} - E_{\pm\frac{1}{2}, 0}^{\text{sec}} \approx E_{\pm\frac{1}{2}, 0}^{\text{sec}} - E_{\pm\frac{1}{2}, -1}^{\text{sec}} \approx 2\pi\nu_{\text{T}} \tag{S22}$$

The second order corrections result in the following eigenvalues:

$$E_{+\frac{1}{2}, +1}^{\text{non-sec}} = E_{+\frac{1}{2}, +1}^{\text{sec}} + \frac{\left| \langle +\frac{1}{2}, +1 | \hat{H}^{\text{non-sec}} | +\frac{1}{2}, 0 \rangle \right|^2}{2\pi\nu_{\text{T}}} = \frac{|E_{\text{ZFS}}^{\text{non-sec}}|^2 + 2\text{Re}(E_{\text{ZFS}}^{\text{non-sec}}) \cdot E_{\text{d}}^{\text{non-sec}} + E_{\text{d}}^{\text{non-sec}^2}}{2\pi\nu_{\text{T}}}, \tag{S23}$$

$$E^{\text{non-sec}}_{+\frac{1}{2},0} = E^{\text{sec}}_{+\frac{1}{2},0} - \frac{\left|\langle +\frac{1}{2},+1|\hat{H}^{\text{non-sec}}|+\frac{1}{2},\ 0\rangle\right|^2}{2\pi\nu_{\text{T}}} + \frac{\left|\langle +\frac{1}{2},0|\hat{H}^{\text{non-sec}}|+\frac{1}{2},\ -1\rangle\right|^2}{2\pi\nu_{\text{T}}} = -4\,\frac{E^{\text{non-sec}}_{\text{ZFS}}\cdot E^{\text{non-sec}}_{\text{dip}}}{2\pi\nu_{\text{T}}}, \tag{S24}$$

$$E^{\text{non-sec}}_{+\frac{1}{2},-1} = E^{\text{sec}}_{+\frac{1}{2},-1} - \frac{\left|\langle +\frac{1}{2},0|\hat{H}^{\text{non-sec}}|+\frac{1}{2},-1\rangle\right|^2}{2\pi\nu_{\text{T}}} = -\frac{\left|E^{\text{non-sec}}_{\text{ZFS}}\right|^2 - 2\text{Re}\left(E^{\text{non-sec}}_{\text{ZFS}}\right)\cdot E^{\text{non-sec}}_{\text{dip}} + E^{\text{non-sec}\,2}_{\text{dip}}}{2\pi\nu_{\text{T}}}, \tag{S25}$$

$$E^{\text{non-sec}}_{-\frac{1}{2},+1} = E^{\text{sec}}_{-\frac{1}{2},+1} + \frac{\left|\langle -\frac{1}{2},+1|\hat{H}^{\text{non-sec}}|-\frac{1}{2},\ 0\rangle\right|^2}{2\pi\nu_{\text{T}}} = \frac{|E^{\text{non-sec}}_{\text{ZFS}}|^2 - 2\text{Re}\left(E^{\text{non-sec}}_{\text{ZFS}}\right)\cdot E^{\text{non-sec}}_{\text{dip}} + E^{\text{non-sec}\,2}_{\text{dip}}}{2\pi\nu_{\text{T}}}, \tag{S26}$$

$$E^{\text{non-sec}}_{-\frac{1}{2},0} = E^{\text{sec}}_{-\frac{1}{2},0} - \frac{\left|\langle -\frac{1}{2},+1|\hat{H}^{\text{non-sec}}|-\frac{1}{2},\ 0\rangle\right|^2}{2\pi\nu_{\text{T}}} + \frac{\left|\langle -\frac{1}{2},0|\hat{H}^{\text{non-sec}}|-\frac{1}{2},\ -1\rangle\right|^2}{2\pi\nu_{\text{T}}} = 4\,\frac{E^{\text{non-sec}}_{\text{ZFS}}\cdot E^{\text{non-sec}}_{\text{dip}}}{2\pi\nu_{\text{T}}}, \tag{S27}$$

$$E^{\text{non-sec}}_{-\frac{1}{2},-1} = E^{\text{sec}}_{-\frac{1}{2},-1} - \frac{\left|\langle -\frac{1}{2},0|\hat{H}^{\text{non-sec}}|-\frac{1}{2},-1\rangle\right|^2}{2\pi\nu_{\text{T}}} = -\frac{\left|E^{\text{non-sec}}_{\text{ZFS}}\right|^2 + 2\text{Re}\left(E^{\text{non-sec}}_{\text{ZFS}}\right)\cdot E^{\text{non-sec}}_{\text{dip}} + E^{\text{non-sec}\,2}_{\text{dip}}}{2\pi\nu_{\text{T}}}. \tag{S28}$$

Here, Re stands for the real-part of a number. The LaserIMD frequencies can then be calculated as follows:

$$\omega^{\text{non-sec}}_{+1} = \left(E^{\text{non-sec}}_{+\frac{1}{2},+1} - E^{\text{non-sec}}_{-\frac{1}{2},+1}\right) - \left(E_{+\frac{1}{2},\text{dark}} - E_{-\frac{1}{2},\text{dark}}\right) = \left(3\cos\left(\beta_{\text{dip}}\right)^2 - 1\right)\omega_{\text{d}} + \frac{4\text{Re}\left(E^{\text{non-sec}}_{\text{ZFS}}\right)\cdot E^{\text{non-sec}}_{\text{dip}}}{2\pi\nu_{\text{T}}}, \tag{S29}$$

$$\omega^{\text{non-sec}}_{0} = \left(E^{\text{non-sec}}_{+\frac{1}{2},0} - E^{\text{non-sec}}_{-\frac{1}{2},0}\right) - \left(E_{+\frac{1}{2},\text{dark}} - E_{-\frac{1}{2},\text{dark}}\right) = -\frac{8\text{Re}\left(E^{\text{non-sec}}_{\text{ZFS}}\right)\cdot E^{\text{non-sec}}_{\text{dip}}}{2\pi\nu_{\text{T}}}, \tag{S30}$$

$$\omega^{\text{non-sec}}_{-1} = \left(E^{\text{non-sec}}_{+\frac{1}{2},-1} - E^{\text{non-sec}}_{-\frac{1}{2},-1}\right) - \left(E_{+\frac{1}{2},\text{dark}} - E_{-\frac{1}{2},\text{dark}}\right) = -\left(3\cos\left(\beta_{\text{dip}}\right)^2 - 1\right)\omega_{\text{dip}} + \frac{4\text{Re}\left(E^{\text{non-sec}}_{\text{ZFS}}\right)\cdot E^{\text{non-sec}}_{\text{dip}}}{2\pi\nu_{\text{T}}}. \tag{S31}$$

Insertion of Eq. (S16) an (S17) for $E^{\text{non-sec}}_{\text{ZFS}}$ and $E^{\text{non-sec}}_{\text{dip}}$ gives the expressions of Eq. (21) –(23) of the main text.

The same corrections of the energy levels can be used to calculate the LiDEER frequencies:

$$\omega^{\text{non-sec}}_{+\frac{1}{2}\to-\frac{1}{2},+1\leftrightarrow0} = \left(E^{\text{non-sec}}_{+\frac{1}{2},+1} - E^{\text{non-sec}}_{+\frac{1}{2},0}\right) - \left(E^{\text{non-sec}}_{-\frac{1}{2},+1} - E^{\text{non-sec}}_{-\frac{1}{2},0}\right) = \left(3\cos\left(\beta_{\text{dip}}\right)^2 - 1\right)\omega_{\text{dip}} + \frac{12\text{Re}\left(E^{\text{non-sec}}_{\text{ZFS}}\right)\cdot E^{\text{non-sec}}_{\text{dip}}}{2\pi\nu_{\text{T}}}, \tag{S32}$$

$$\omega^{\text{non-sec}}_{+\frac{1}{2}\to-\frac{1}{2},+1\leftrightarrow0} = \left(E^{\text{non-sec}}_{+\frac{1}{2},+1} - E^{\text{non-sec}}_{+\frac{1}{2},0}\right) - \left(E^{\text{non-sec}}_{-\frac{1}{2},+1} - E^{\text{non-sec}}_{-\frac{1}{2},0}\right) = -\left(3\cos\left(\beta_{\text{dip}}\right)^2 - 1\right)\omega_{\text{dip}} + \frac{12\text{Re}\left(E^{\text{non-sec}}_{\text{ZFS}}\right)\cdot E^{\text{non-sec}}_{\text{dip}}}{2\pi\nu_{\text{T}}}, \tag{S33}$$

$$\omega^{\text{non-sec}}_{+\frac{1}{2}\to-\frac{1}{2},0\leftrightarrow-1} = \left(E^{\text{non-sec}}_{+\frac{1}{2},+1} - E^{\text{non-sec}}_{+\frac{1}{2},0}\right) - \left(E^{\text{non-sec}}_{-\frac{1}{2},+1} - E^{\text{non-sec}}_{-\frac{1}{2},0}\right) = \left(3\cos\left(\beta_{\text{dip}}\right)^2 - 1\right)\omega_{\text{dip}} - \frac{12\text{Re}\left(E^{\text{non-sec}}_{\text{ZFS}}\right)\cdot E^{\text{non-sec}}_{\text{dip}}}{2\pi\nu_{\text{T}}}, \tag{S34}$$

$$\omega^{\text{non-sec}}_{+\frac{1}{2}\to-\frac{1}{2},0\leftrightarrow-1} = \left(E^{\text{non-sec}}_{+\frac{1}{2},+1} - E^{\text{non-sec}}_{+\frac{1}{2},0}\right) - \left(E^{\text{non-sec}}_{-\frac{1}{2},+1} - E^{\text{non-sec}}_{-\frac{1}{2},0}\right) = -\left(3\cos\left(\beta_{\text{dip}}\right)^2 - 1\right)\omega_{\text{dip}} - \frac{12\text{Re}\left(H^{\text{non-sec}}_{\text{ZFS}}\right)\cdot H^{\text{non-sec}}_{\text{dip}}}{2\pi\nu_{\text{T}}}. \tag{S35}$$

5

S2 Influence of the remaining non-secular terms

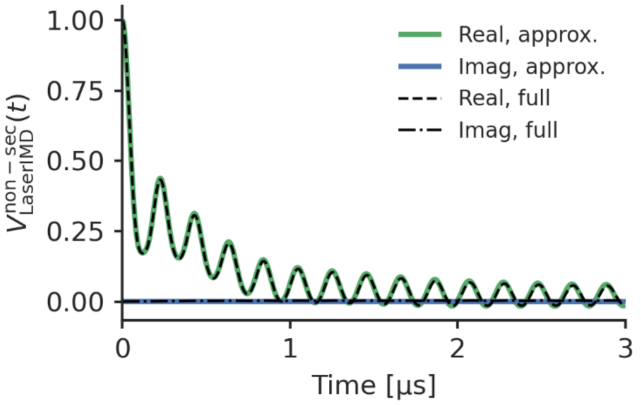

Figure S1: The dotted lines show a numerical calculation of a LaserIMD trace as a numerical simulation of the Liouville-von-Neumann equation with all terms in the Hamiltonian, the green and blue trace show the same trace calculated with the equations, derived in SI1. Both simulations used the values: $D = 1159$ MHz, $E = -238$ MHz, $P_x = 0.33$, $P_y = 0.41$, $P_z = 0.26$, $r = 2.2$ nm and $\nu_T = 9.3$ GHz .

S3 Convergence of the integral

For the test-run, the following parameters were used: D = 1159 MHz, E= −238 MHz, P_x = 0.33, P_y = 0.41, P_z = 0.26, r = 2.2 nm and ν_T = 9.3 GHz. For the dipolar grid, the relative change was below 1 % when going from 100 to 200 grid points. In Figure S2 the convergence in dependence of the number of grid points for the transient label is shown. Whereas $V_{+1}^{\text{non-sec}}(t)$ and $V_{-1}^{\text{non-sec}}(t)$ have already converged when going from 100 to 200 points for the grid of the transient label, $V_{\text{LaserIMD}}^{\text{non-sec}}(t)$ and $V_0^{\text{non-sec}}(t)$ converge much slower. Particularly $V_0^{\text{non-sec}}(t)$ requires 12800 points until it is sufficiently converged. Figure S3 shows how $V_0^{\text{non-sec}}(t)$ changes when the number of grid points is increased.

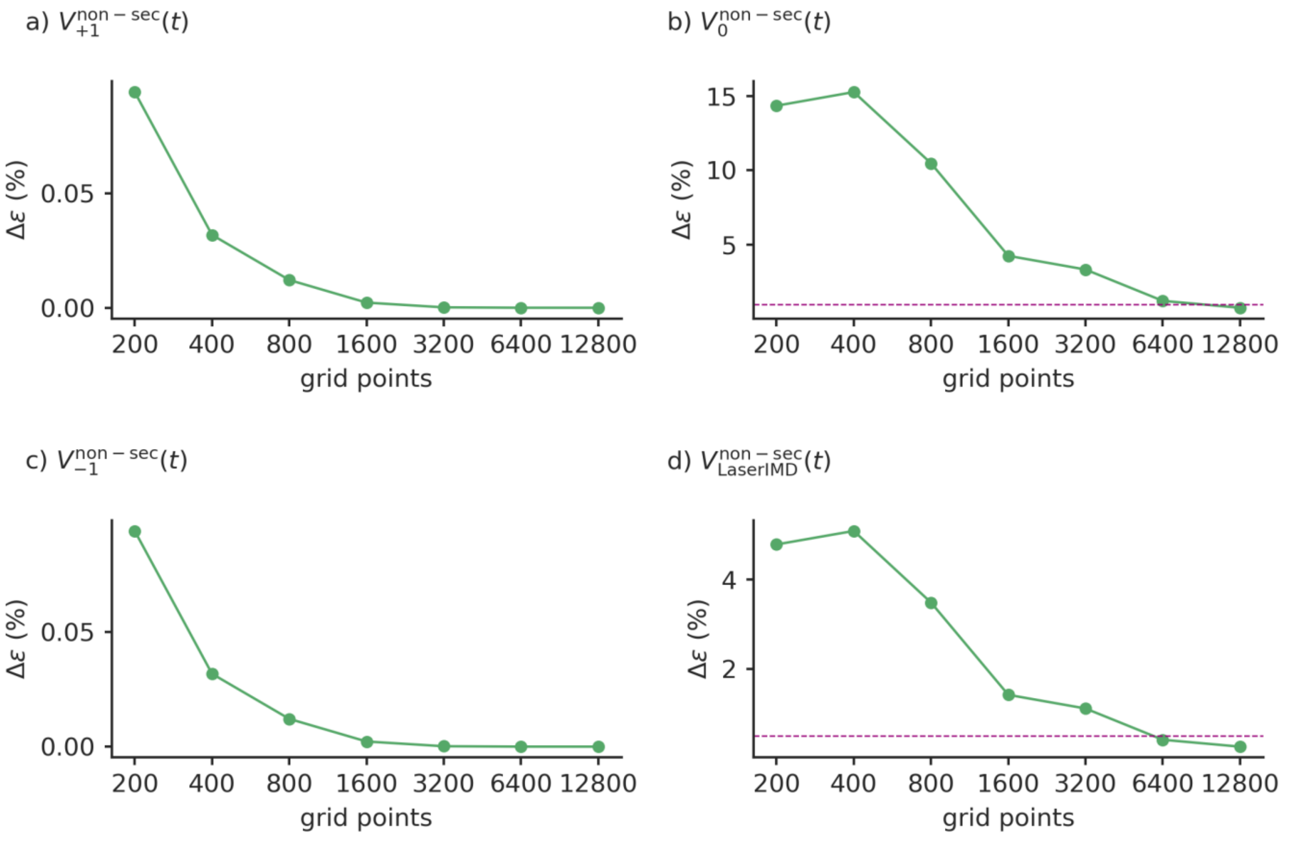

Figure S2: The relative change of the numerically calculated integral as a function of increasing grid points for a) $V_{+1}^{\text{non-sec}}(t)$, b) $V_0^{\text{non-sec}}(t)$, c) $V_{-1}^{\text{non-sec}}(t)$, d) $V_{\text{LaserIMD}}^{\text{non-sec}}(t) = V_{+1}^{\text{non-sec}}(t) + V_0^{\text{non-sec}}(t) + V_{-1}^{\text{non-sec}}(t)$. The parameters are set to D = 1159 MHz, E= −238 MHz, P_x = 0.33, P_y = 0.41, P_z = 0.26, r = 2.2 nm and ν_T = 9.3 GHz. The grid for the dipolar vector contained 200 points. The 1 % line is drawn in b) and d), for the a) and c) it is outside the boundaries of the plot. The relative change at 200 points refers to the signals that were calculated with 100 pts.

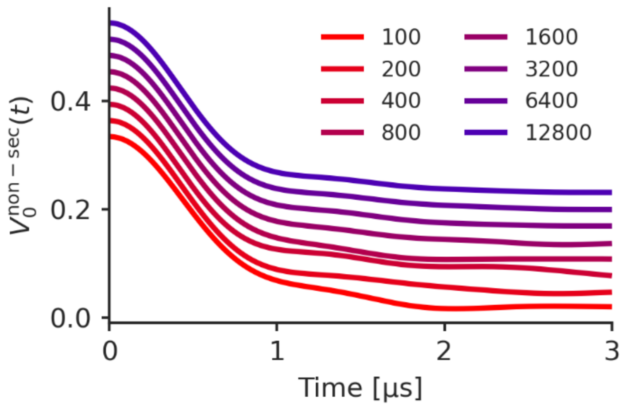

Figure S3: The numerical integration for $V_0^{\mathrm{non-sec}}(t)$ for different number of points for the grid of the triplet. $D = 1159$ MHz, E$=-238$ MHz, $P_x = 0.33$, $P_y = 0.41$, $P_z = 0.26$, r $= 2.2$ nm and $\nu_{\mathrm{T}} = 9.3$ GHz. The grid for the dipolar vector contained 200 points. The traces are shifted by 0.03 for better visibility.

S4 LaserIMD simulations for different Zeeman frequencies

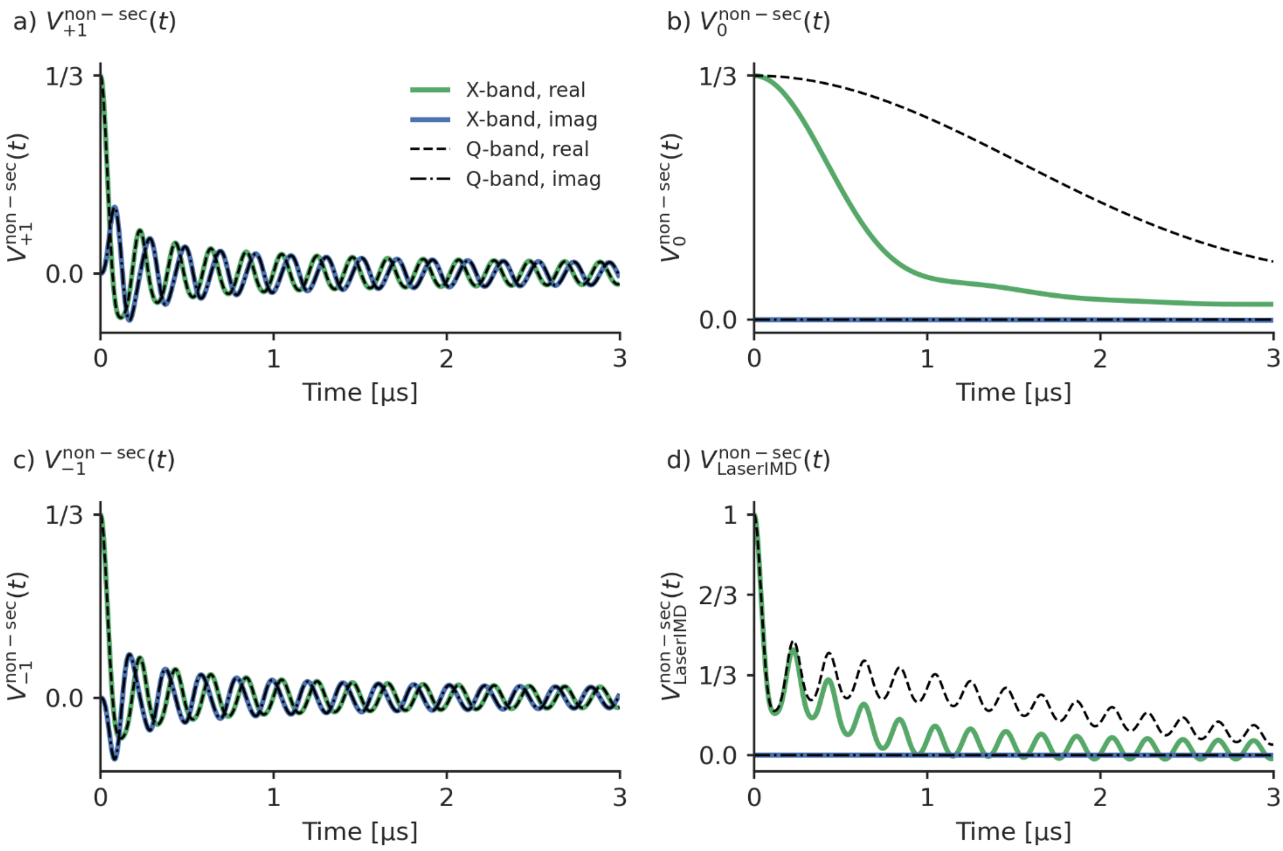

Figure S4: Simulations with TPP as transient spin label and $r = 2.2$ nm and $\nu_{\mathrm{T}} = 34$ GHz (green and blue) and $\nu_{\mathrm{T}} = 9.3$ GHz (black dashed lines). a) $V_{+1}^{\mathrm{non-sec}}(t)$, b) $V_{0}^{\mathrm{non-sec}}(t)$, c) $V_{-1}^{\mathrm{non-sec}}(t)$, d) $V_{\mathrm{LaserIMD}}^{\mathrm{non-sec}}(t) = V_{+1}^{\mathrm{non-sec}}(t) + V_{0}^{\mathrm{non-sec}}(t) + V_{-1}^{\mathrm{non-sec}}(t)$.

S5 LaserIMD simulations for different ZFS values

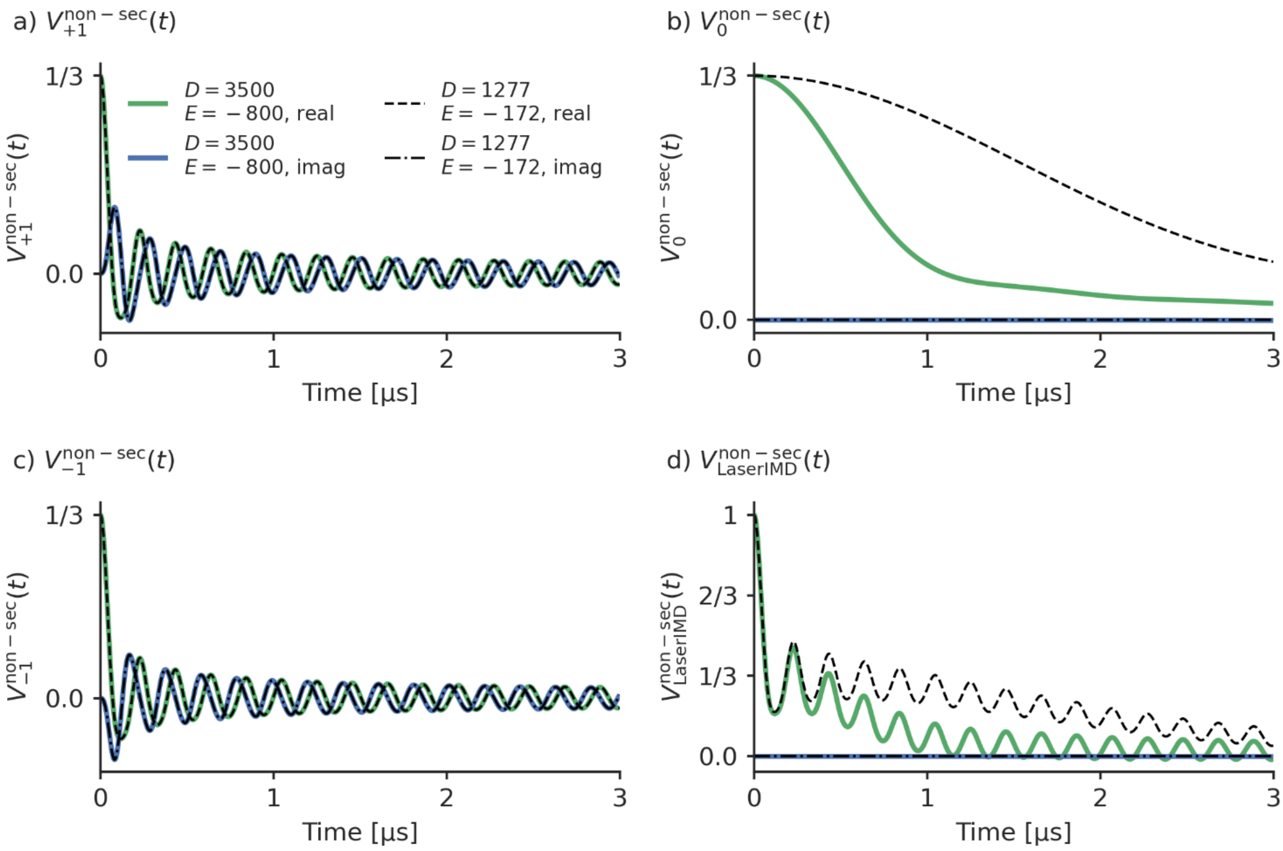

Figure S5: Simulations with different ZFS values with the following parameters: $P_x = 0.33$, $P_y = 0.41$, $P_z = 0.26$, $r = 2.2$ nm, $\nu_{\mathrm{T}} = 9.3$ GHz and $D = 1227$ MHz, E= -172 MHz (green and blue) and $D = 3500$ MHz, E= -800 MHz (black dashed lines) a) $V_{+1}^{\mathrm{non-sec}}(t)$, b) $V_{0}^{\mathrm{non-sec}}(t)$, c) $V_{-1}^{\mathrm{non-sec}}(t)$, d) $V_{\mathrm{LaserIMD}}^{\mathrm{non-sec}}(t) = V_{+1}^{\mathrm{non-sec}}(t) + V_{0}^{\mathrm{non-sec}}(t) + V_{-1}^{\mathrm{non-sec}}(t)$. The ZFS values in the legend are given in MHz.

S6 LaserIMD simulations for different distances

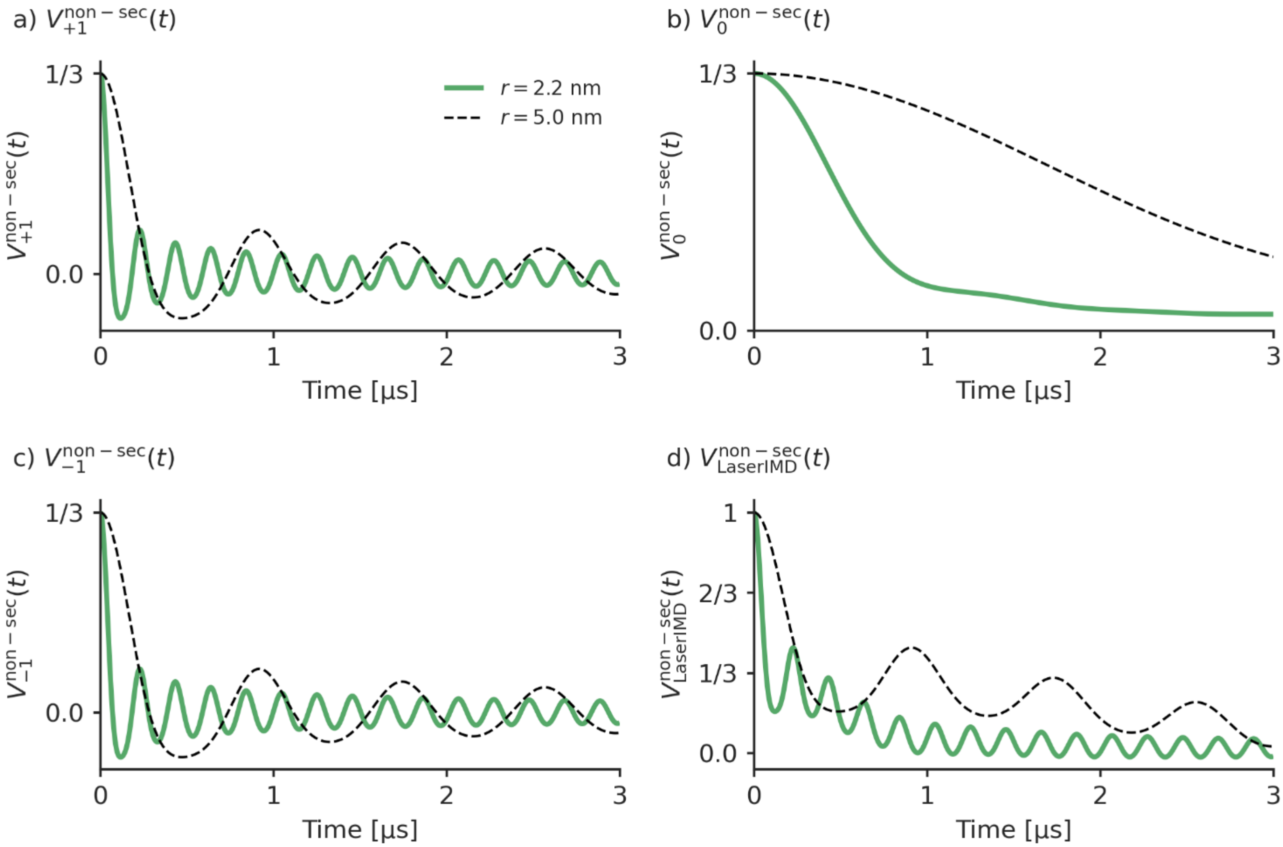

Figure S6: Simulations with TPP as transient spin label and ν_T = 9.3 GHz and r = 2.2 nm (green) and r = 5.0 nm (dashed black lines). a) $V_{+1}^{\mathrm{non-sec}}(t)$, b) $V_{0}^{\mathrm{non-sec}}(t)$, c) $V_{-1}^{\mathrm{non-sec}}(t)$, d) $V_{\mathrm{LaserIMD}}^{\mathrm{non-sec}}(t) = V_{+1}^{\mathrm{non-sec}}(t) + V_{0}^{\mathrm{non-sec}}(t) + V_{-1}^{\mathrm{non-sec}}(t)$.

S7 Calculation of distance distributions with Tikhonov regularization

The distance distributions of simulated and experimentally recorded traces were obtained with the package *DeerLab* (version 0.13.2) (Fábregas Ibáñez et al., 2020) in *Python* 3.9. As a first step, a phase- and zero-time correction was done. The traces were then fitted with Tikhonov regularization in a one-step procedure, where the background and distance distribution are not fitted subsequently but in one step. The dipolar-kernel of a four-pulse DEER experiment $K_{\mathrm{DEER}}(t, r)$ was used and the background function was assumed to have the shape of an exponential decay. The regularization parameter was chosen according to the Akaike information criterion (Edwards and Stoll, 2018). The validation was performed with bootstrapping by generating 1000 sampled with artificial noise that were subsequently analyzed. The error was then calculated as the 95 % confidence interval.

S8 LiDEER simulations

The time-domain simulations of the LiDEER pulse sequence laser pulse – DAF – $\pi/2$ – τ_1 – π – t – π – pump – (τ_2 – t) – π – τ_{delay} – echo (Di Valentin et al., 2014) were performed with the package *Spinach* (version 2.6.5625) (Hogben et al., 2011) in MATLAB (R2021b). *Spinach* calculates a numerical solution of the Liouville-von-Neumann equation by density matrix propagation. The spin system consisted of a photoexcited transient spin label in its triplet state and a permanent spin label that was included as a doublet spin. For the transient spin label we chose TPP or a spin system with a larger ZFS ($D = 3500$ MHz, $E = -800$ MHz) and simulations in X- and Q-band were performed. No additional hyperfine interactions were included. The distance between the two spins was set to $r = 2.2$ nm. The full spin Hamiltonian including all terms from Eq. 10 from the main text was used, and the simulations were performed in the laboratory frame. The starting density matrix was obtained as the Kronecker product of the spin polarized state of the transient label which was calculated according to (Williams et al., 2020) and the equilibrated state of the permanent spin label ($T = 30$ K). All operators and states were treated in Liouville representation with spherical tensors. The effects of the microwave pulses with a finite width and power were calculated by stepwise propagation in the time domain. The power of the microwave pulses was calculated such that the flip angles corresponds to the required $\pi/2$ or π and for the transient spin label, the effect of the higher transition dipole moment was considered. The observer frequency was set to be either be on-resonance to the Y^+ canonical peak or it was set to a part of the spectrum between the X^+ and the Y^+ peak where all canonical orientations are off-resonance. The pump frequency was set to the frequency of the permanent radical (see Figure S7). The signal was obtained by summing over the echo in the range of the full-width half maximum. This integration window and the exact time delay τ_{delay} of the echo were determined manually by the simulation of the echo. The powder average was done by summing over the different orientations for the transient spin label and the dipolar coupling vector. For that, the same grids as for the LaserIMD simulations were used. As the permanent spin label does not contain any anisotropic interaction, no powder averaging was performed for it. The source code for the LiDEER simulations can be downloaded at https://github.com/andreas-scherer/LiDEER_simulations.git.

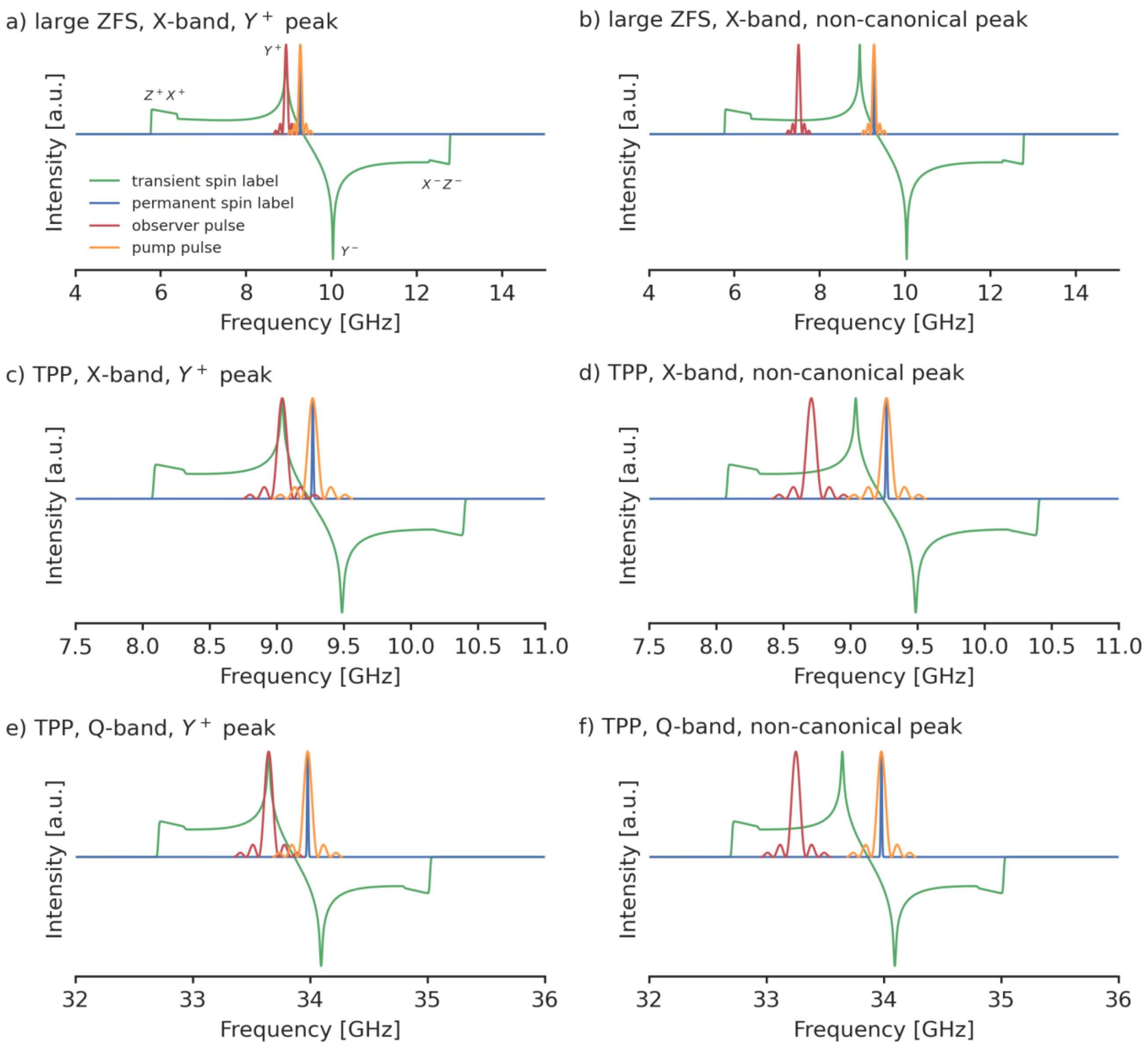

Figure S7: The EPR spectra of the transient spin label (green) with the canonical peaks marked in a), the permanent spin label (blue), the excitation profile of the observer π-pulse (red) and the pump π-pulse (orange) that were used in the LiDEER simulations. a) and b) $D = 3500$ MHz, $E = -800$ MHz, $P_x = 0.33$, $P_y = 0.41$ and $P_z = 0.26$ in X-band; c) and d) TPP as transient spin label in X-band; e) and f) TPP as transient spin label in Q-band. a), c) and e) the observer pulse is positioned on the Y^+ peak. b), d) and f) the observer pulse is positioned off-resonance to all canonical orientations. The EPR spectra and excitation profiles were simulated with *easyspin* (Stoll and Schweiger, 2006). The relative intensities of the EPR spectra of the transient and permanent spin labels do not reflect their actual intensities. They are both normalized for better visibility.

Table S1: The details of the time-domain LiDEER simulations.

	**Y^+ orientation**			**Non-canonical orientation**		
	**Large ZFS (X-band)**	**TPP (X-band)**	**TPP (Q-band)**	**Large ZFS (X-band)**	**TPP (X-band)**	**TPP (Q-band)**
**g_D-value (permanent spin label)**	2			2		
**g_T-value (transient spin label)**	2.00687			2.00687		
**D/E [MHz]**	3500/ -800	1159/ -238		3500/ -800	1159/ -238	
**$P_x/P_y/P_z$**	0.33/ 0.41/ 0.26			0.33/ 0.41/ 0.26		
**Magnetic field [T]**	0.33		1.2097	0.33		1.2097
**Observer frequency [GHz]**	8.934	9.042	33.646	7.500	8.709	3.250
**Pump frequency [GHz]**	9.269		33.979	9.269		33.979
**Observer $\pi/2$-pulse length [ns]**	10			20		
**Observer π-pulse length [ns]**	20			20		
**Pump π-pulse length [ns]**	10			10		
**Observer $\pi/2$-pulse power [MHz]**	17.68			17.68		
**Observer π-pulse power [MHz]**	17.68			17.68		
**Pump π-pulse power [MHz]**	50			50		
**τ_1 [μs]**	0.1			0.1		
**τ_2 [μs]**	2.1			2.1		
**Time step $\Delta\tau$ [ns]**	8			8		

S9 Results of the LiDEER simulations on a non-canonical orientation

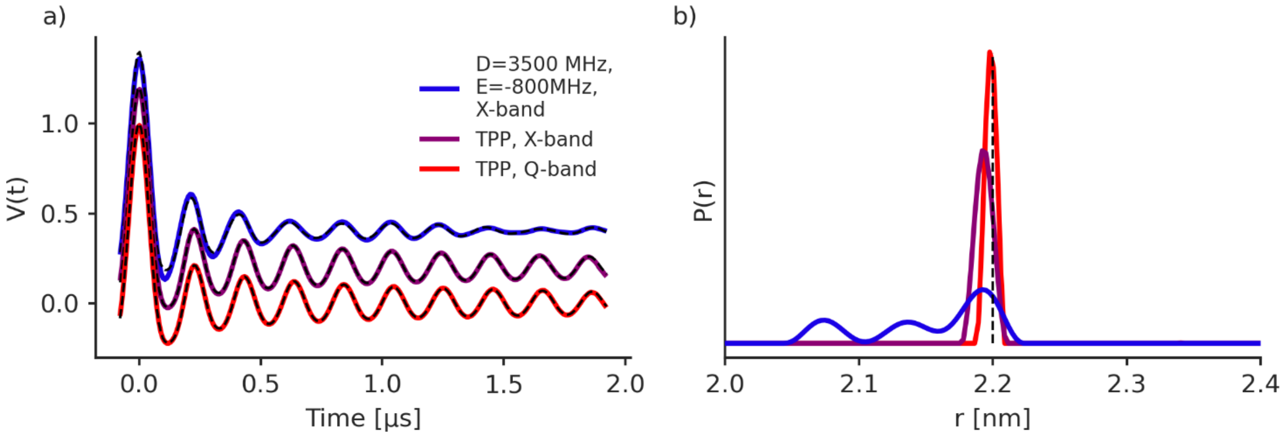

Figure S8: a) Numerical simulation of LiDEER with the observer pulse placed off-resonance to all canonical peaks of the EPR spectrum of the transient spin label at different frequency bands and with different ZFS. The traces are shifted by 0.2 for better visibility. For Q-band and TPP the magnetic field was set to 1.2097 T and the observer frequency to 33.250 GHz, for X-band the magnetic field was set to 0.33 T, which for TPP corresponds to an observer frequency of 8.709 GHz and for a ZFS with D = 3500 MHz and E = −800 MHz to an observer frequency of 7.500 GHz. The position of the observer and pump pulse with respect to the EPR spectrum is shown in Fig. S7b, d and f. The further parameters are $P_x = 0.33$, $P_y = 0.41$, $P_z = 0.26$ and $r = 2.2$ nm. The numerical simulations were fitted with Tikhonov regularization and a dipolar kernel that does not take non-secular interactions into account. The fits are shown as black line and b) shows the corresponding distance distribution. The true distance of $r = 2.2$ nm is plotted as dotted black line.

S10 EPR experiments

Sample preparation

All solvents were purchased from Merck and used without further purification. The peptides TPP-pAA$_5$-NO• and TPP-pAA$_{10}$-NO• were purchased from Biosynthan as freeze-dried powder and used without further purification. They were dissolved in 98/2 MeOD$_4$/ D$_2$O [vol.%]. The TPP-pAA$_5$-NO• sample had a concentration of 10 µM and the TPP-pAA$_{10}$-NO• sample had a concentration of 50 µM. The samples were loaded in a 3mm outer-diameter quartz-tube. A sample volume of 10 µl (filling height 5 mm) was used in order to maintain a uniform excitation throughout the sample. Prior to shock-freezing in liquid nitrogen, these samples were also degassed by three consecutive freeze-pump-thaw cycles for oxygen removal. All samples were measured at a temperature of 30 K with a repetition time of 50 ms. The laser excitation took place at a wavelength of 510 nm.

The laser system

Light excitation was achieved with a tunable diode pumped Nd:YAG laser system NT230-50-ATTN2-FC (Ekspla, Vilnius, Lithuania) comprising a pump laser, second and third harmonic generators and an optical parametric oscillator (OPO). Unless stated otherwise, the system was operated with pulse energies of ≈ 3.5 mJ (measured before the laser fiber, standard deviation ≈ 0.2 µJ). As the fibre has a transmission of approximately 40 %, this results in an energy after the fiber of ≈ 1.4 mJ. Triggering was performed by means of the spectrometer's pulse PatternJet (Q-band pulsed EPR measurements) or an external pulse generator (time resolved EPR measurements). The light was coupled into the resonator using a quartz glass fiber (1 mm core, Pigtail WF 1000/ 1100/ 1600 T, CeramOptec GmbH, Bonn, Germany), with its end adjusted to a height of 1 cm above the sample surface.

X-band measurements

X-band EPR spectra were recorded on a Bruker Elexsys E580 spectrometer (Bruker Biospin) and a 1 kW amplifier in an overcoupled ER4118X-MS3 resonator (Bruker Biospin). It was critically coupled to a Q-value of ≈ 900-2000. The temperature was controlled with a CF935 helium gas flow system (Oxford instruments) controlled by an ITC (Oxford Instruments).

Q-band measurements

Q-band measurements were performed on a Bruker Elexsys E580 spectrometer equipped with a SpinJet-AWG unit (Bruker Biospin, Rheinstetten, Germany) and a 150 W pulsed TWT amplifier (Applied Systems Engineering, Fort Worth, USA). A commercial Q-band resonator from Bruker Biospin (ER5106QT-2) was used for all pulsed EPR measurements. It was overcoupled to a Q-factor of ≈ 200. The temperature was maintained with the EPR Flexline helium recirculation system (CE-FLEX-4K-0110, Bruker Biospin, ColdEdge Technologies), comprising a cold head (expander, SRDK-408D2) and a F-70H compressor (both SHI cryogenics, Tokyo, Japan), controlled by an Oxford Instruments Mercury ITC. Echo signals were

detected in integrator mode with a video bandwidth of 20 MHz. The integrator gate was set symmetrically around the echo with a length that equals the full width at half maximum of the echo.

LiDEER

LiDEER measurements were performed in Q-band with the pulse-sequence: laser pulse – DAF – $\pi/2 - \tau_1 - \pi - t - \pi_{pump} - (\tau_1 + \tau_2 - t) - \pi - \tau_2$ – echo (Di Valentin et al., 2014) (note that due to technical reasons the definitions of some of the parameters in the LiDEER pulse-sequence for the experiments are different to those that were used for the time-domain simulations). The delay-after-flash (DAF) was set to 500 ns and τ_1 to 400 ns. Nuclear modulation averaging was performed by varying the τ_1 time in 8 steps with $\Delta\tau_1 = 16$ ns. Phase cycling was performed with the 8-step scheme ((x) [x] xp x) as proposed by (Tait and Stoll, 2016). The observer frequency was set to 34.00 GHz and the pump frequency to 33.84 GHz. All pulses were rectangular pulses. All further parameters can be found in Table S2.

Table S2: The parameters for the LiDEER measurements.

	**TPP-pAA$_5$-NO•**	**TPP-pAA$_{10}$-NO•**
**Magnetic field [T]**	1.2045	1.2052
**Observer π-pulse length [ns]**	20	20
**Pump π-pulse length [ns]**	48	52
**τ_1 [µs]**	0.4	0.4
**Trace length τ_2 [µs]**	2.6	6.1
**Time step $\Delta\tau$ [ns]**	8	16
**Microwave attenuation [dB]**	0	0
**Shots per point**	1	1
**Number of averages**	74	65
**Video gain [dB]**	21	21

LaserIMD

LaserIMD experiments were performed with the pulse-sequence $\pi/2 - \tau - \pi - t$ - laser pulse - (τ-t) - echo reported by (Hintze et al., 2016). A 2-step phase cycle was implemented for baseline correction. All pulses were rectangular pulses. The magnetic field was set to the maximum of the nitroxide EPR spectrum at the corresponding microwave frequency, All further parameters can be found in Table S3.

Table S3: The parameters for the LaserIMD measurements.

	TPP-pAA$_5$-NO•		TPP-pAA$_{10}$-NO•	
**Microwave frequency [GHz]**	9.28 (X-band)	34.0 (Q-band)	9.31 (X-band)	34.0 (Q-band)
**Magnetic field [T]**	0.3304	1.2097	0.3314	1.2097
**π-pulse length [ns]**	24	28	24	28
**Trace length τ [µs]**	1.4	2.5	3.2	6.0
**Time step $\Delta\tau$ [ns]**	8	8	16	16
**Microwave attenuation [dB]**	13	0	11	0
**Shots per point**	10	10	10	10
**Number of averages**	483	146	346	177
**Video gain [dB]**	50	27	50	36

reLaserIMD

For the determination for the zero-time as described in (Scherer et al., 2022) a refocused LaserIMD (reLaserIMD) trace was recorded in X- and Q-band with TPP-pAA$_5$-NO•. As the zero-time does not depend on the type of sample, the thus determined zero-time was also used for the peptide TPP-pAA$_{10}$-NO•. We used the shorter peptide for zero-time determination, because due to the faster dipolar oscillations it can be determined more precisely. We used the pulse-sequence $\pi/2 - \tau_1 - \pi - t$ - laser pulse - $(\tau_1 + \tau_2 - t) - \tau_2 - \pi$ - echo as reported by (Dal Farra et al., 2019). A 2-step phase cycle was implemented for baseline correction. All pulses were rectangular pulses. The magnetic field was set to the maximum of the nitroxide EPR spectrum at the corresponding microwave frequency. All further parameters can be found in Table S4.

Table S4: The parameters for the reLaserIMD measurements.

	TPP-pAA$_5$-NO•	
**Microwave frequency [GHz]**	9.28 (X-band)	34.0 (Q-band)
**Magnetic field [T]**	0.3304	1.2097
**π-pulse length [ns]**	24	28
**τ_1 [μs]**	0.4	0.8
**Trace length τ_2[μs]**	0.4	1.0
**Time step $\Delta\tau$ [ns]**	8	8
**Microwave attenuation [dB]**	13	0
**Shots per point**	10	10
**Number of averages**	10	219
**Video gain [dB]**	50	27

S11 Analysis of the experimental LaserIMD data

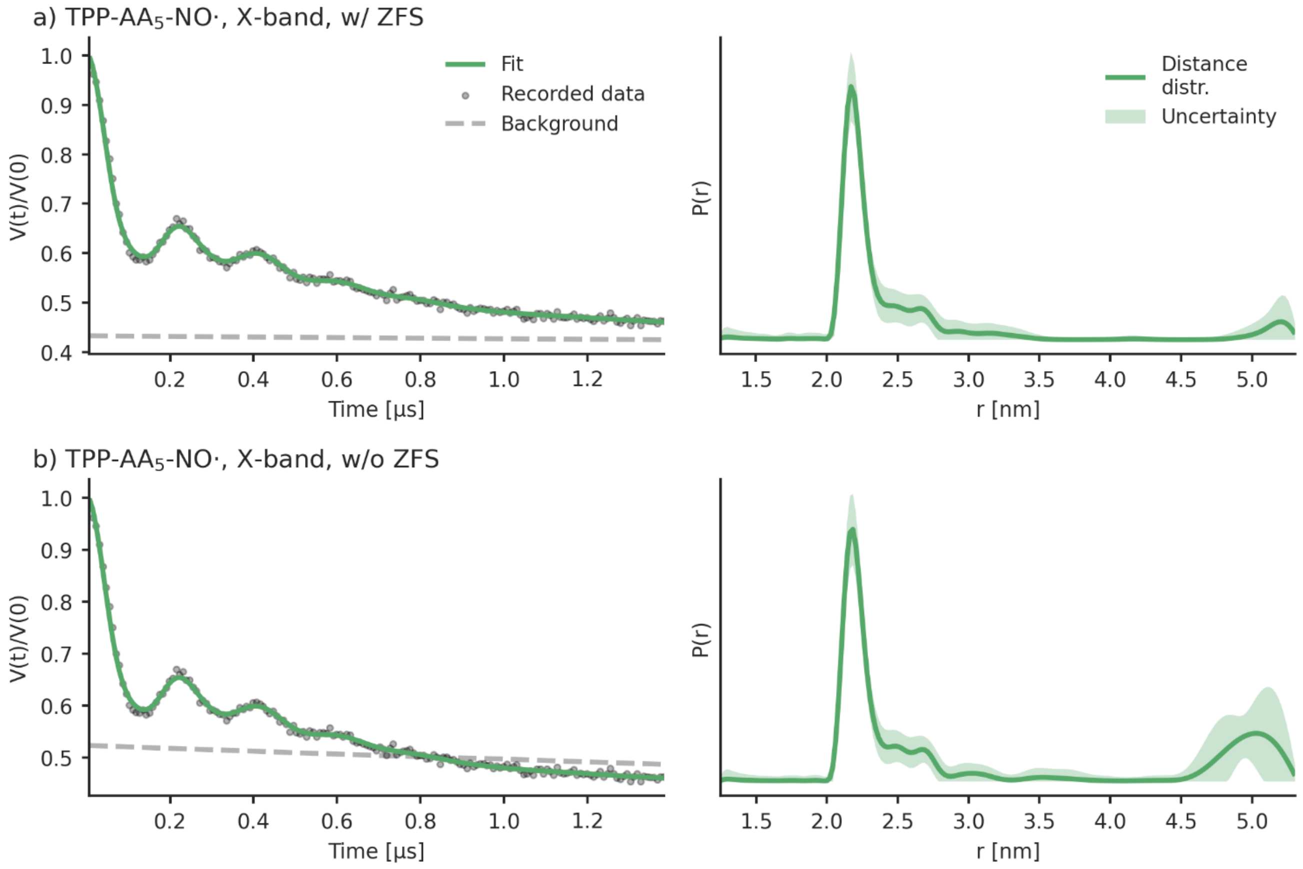

Figure S9: Experimental LaserIMD data of TPP-pAA$_5$-NO• recorded in X-band at 30 K in MeOD/D$_2$O (98/2 vol.%). a) Analyzed with a kernel that includes the ZFS and b) Analyzed with a kernel that ignores the ZFS. The raw data are depicted on the left side as grey dots with the fits as green line, the background fit is depicted as dashed grey line. The distance distributions obtained with Tikhonov regularization (Fábregas Ibáñez et al., 2020) is shown on the right side. The shaded areas correspond to the 95% confidence intervals that were obtained with bootstrapping.

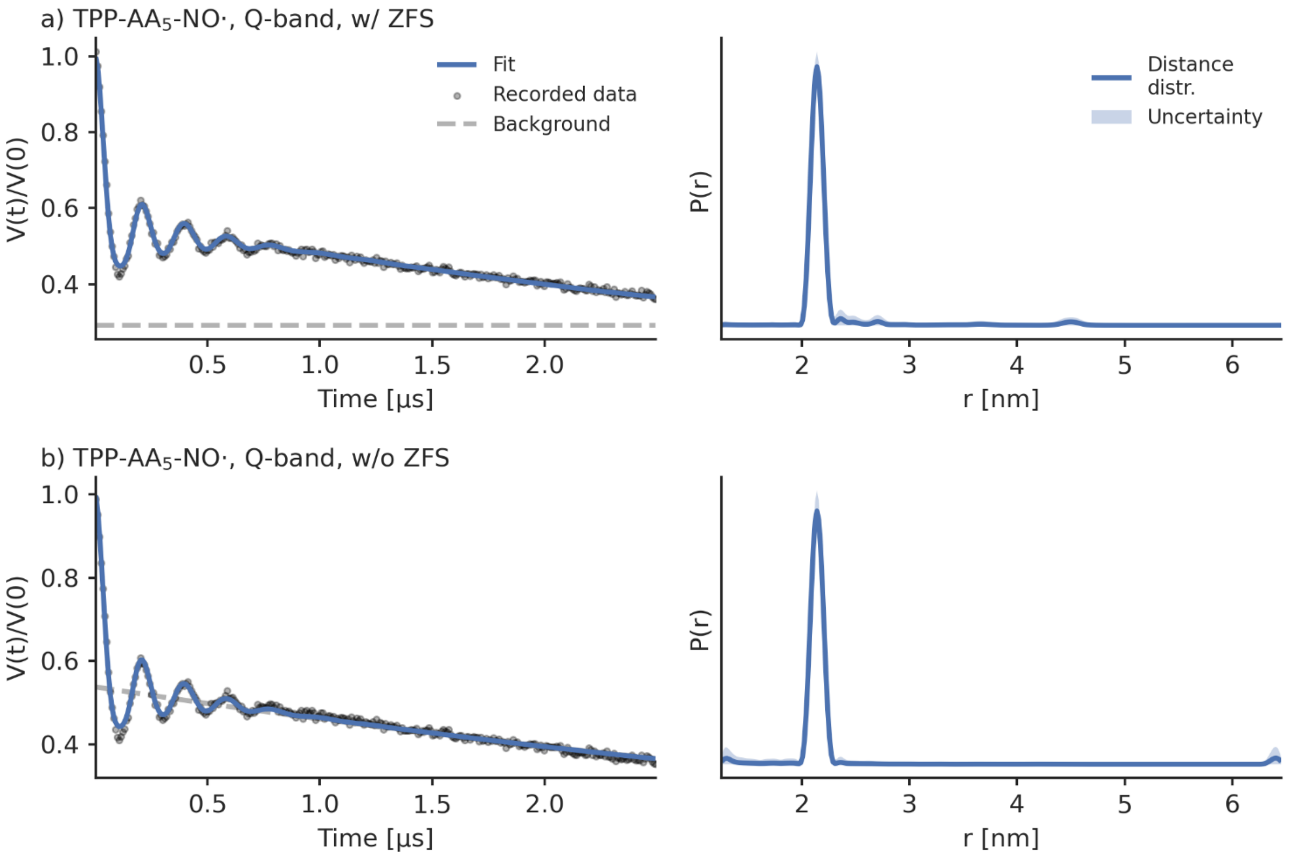

Figure S10: Experimental LaserIMD data of TPP-pAA$_5$-NO• recorded in Q-band at 30 K in MeOD/D$_2$O (98/2 vol.%). a) Analyzed with a kernel that includes the ZFS and b) Analyzed with a kernel that ignores the ZFS. The raw data are depicted on the left side as grey dots with the fits as blue line, the background fit is depicted as dashed grey line. The distance distributions obtained with Tikhonov regularization (Fábregas Ibáñez et al., 2020) is shown on the right side. The shaded areas correspond to the 95% confidence intervals that were obtained with bootstrapping.

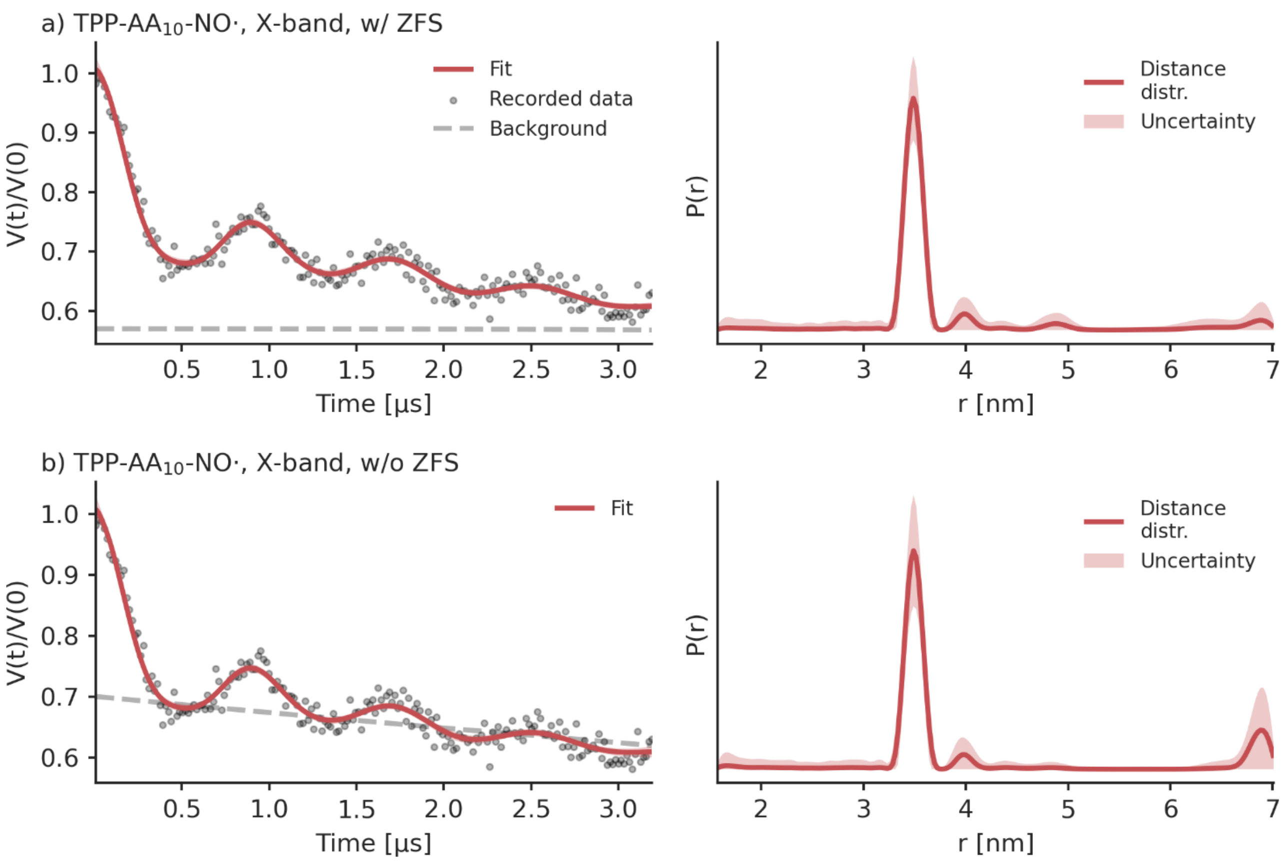

Figure S11: Experimental LaserIMD data of TPP-pAA$_{10}$-NO• recorded in X-band at 30 K in MeOD/D_2O (98/2 vol.%). a) Analyzed with a kernel that includes the ZFS and b) Analyzed with a kernel that ignores the ZFS. The raw data are depicted on the left side as grey dots with the fits as red line, the background fit is depicted as dashed grey line. The distance distributions obtained with Tikhonov regularization (Fábregas Ibáñez et al., 2020) is shown on the right side. The shaded areas correspond to the 95% confidence intervals that were obtained with bootstrapping.

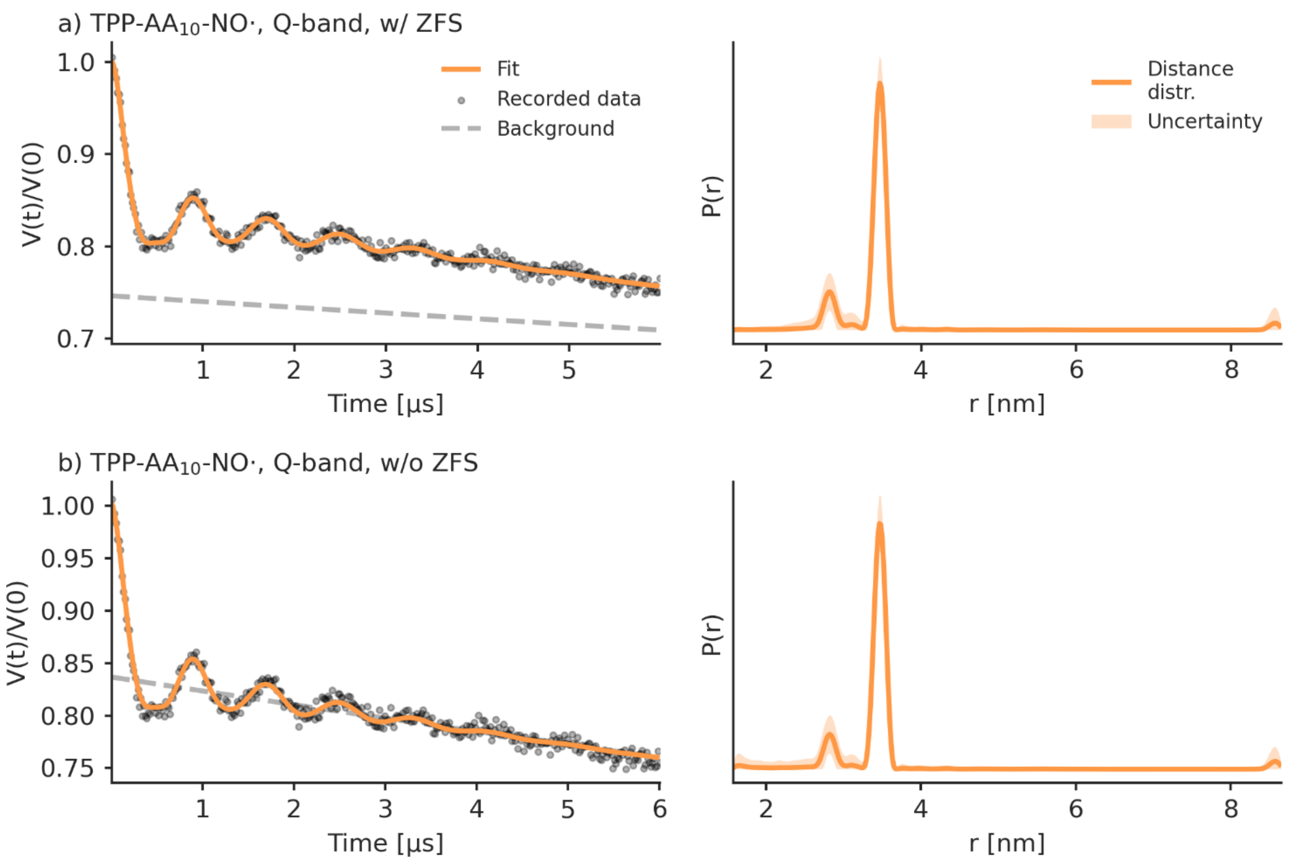

Figure S12: Experimental LaserIMD data of TPP-pAA$_{10}$-NO• recorded in Q-band at 30 K in MeOD/D_2O (98/2 vol.%). a) Analyzed with a kernel that includes the ZFS and b) Analyzed with a kernel that ignores the ZFS. The raw data are depicted on the left side as grey dots with the fits as orange line, the background fit is depicted as dashed grey line. The distance distributions obtained with Tikhonov regularization (Fábregas Ibáñez et al., 2020) is shown on the right side. The shaded areas correspond to the 95% confidence intervals that were obtained with bootstrapping.

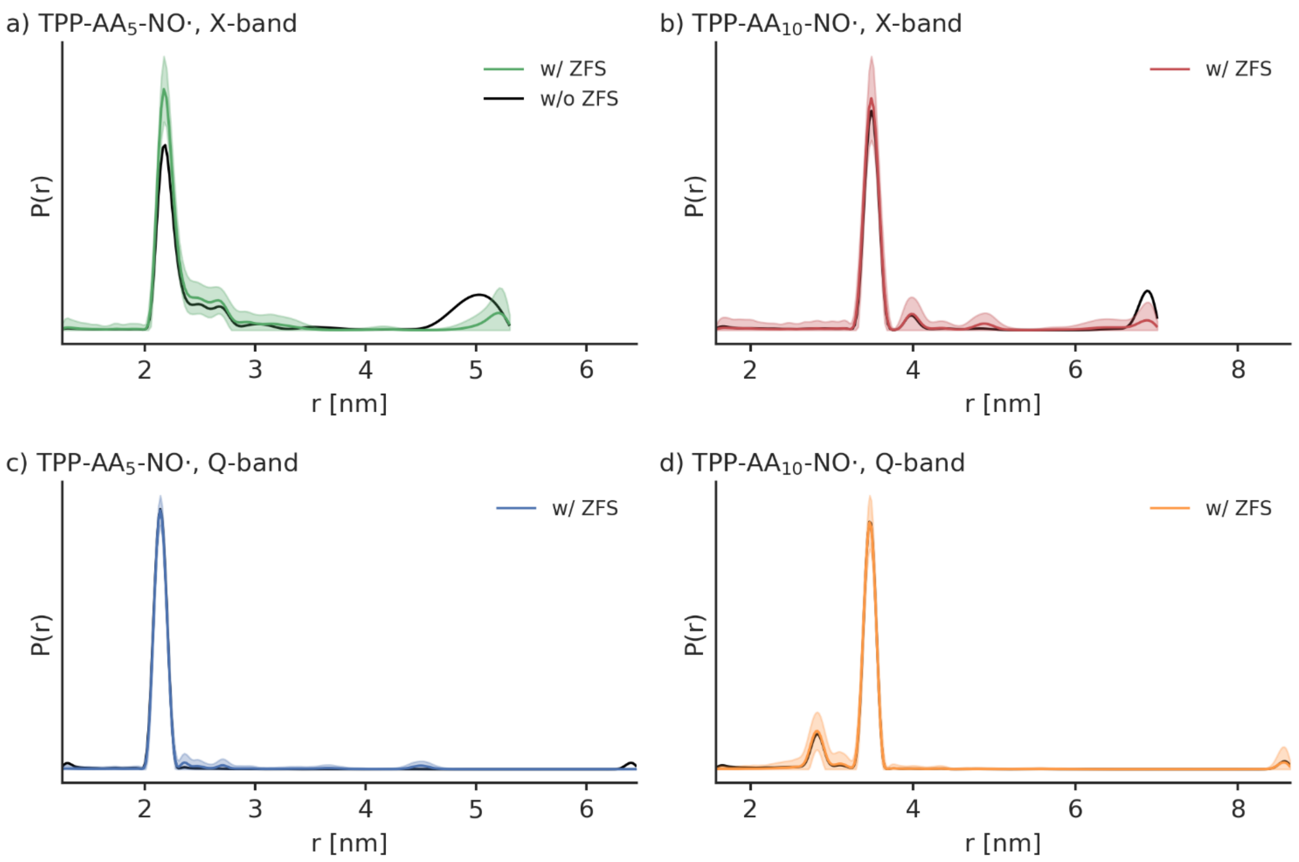

Figure S13: A comparison of the distance distributions that were obtained by analyzing the experimental LaserIMD data with a kernel that includes the ZFS (coloured lines) and with a kernel that ignores the FS (black lines). a) TPP-pAA$_5$-NO• in X-band. b) TPP-pAA$_5$-NO• in Q band. c) TPP-pAA$_{10}$-NO• in X-band. a) TPP-pAA$_{10}$-NO• in Q-band.

Table S5: Background decay rates and modulation depths as obtained by the analysis of the LaserIMD data of TPP-pAA$_5$-NO•.

	**X-band**		**Q-band**	
	w/ ZFS	w/o ZFS	w/ ZFS	w/o ZFS
**Background decay rate [μs^{-1}]**	0.0 (0.0, 0.2)	0.1 (0.0, 0.3)	0.000 (0.003, 0.000)	0.33 (0.27, 0.36)
**Modulation depth [%]**	57 (52, 59)	48 (43, 52)	71 (70, 72)	46 (45, 49)

Table S6: Background decay rates and modulation depths as obtained by the analysis of the LaserIMD data of TPP-pAA$_{10}$-NO•.

	**X-band**		**Q-band**	
	w/ ZFS	w/o ZFS	w/ ZFS	w/o ZFS
**Background decay rate [μs^{-1}]**	0.00 (0.00, 0.05)	0.13 (0.01, 0.28)	0.03 (0.01, 0.04)	0.09 (0.07, 0.11)
**Modulation depth [%]**	43 (40, 45)	31 (25, 36)	26 (25, 27)	17 (16, 18)

S12 References

Abragam, A. and Bleaney, B.: Electron paramagnetic resonance of transition ions, Oxford University Press, 2012.

Blank, A. and Levanon, H.: Triplet line shape simulation in continuous wave electron paramagnetic resonance experiments, Concepts Magn. Reson. Part A, 25A, 18–39, https://doi.org/10.1002/cmr.a.20030, 2005.

Dal Farra, M. G., Richert, S., Martin, C., Larminie, C., Gobbo, M., Bergantino, E., Timmel, C. R., Bowen, A. M., and Di Valentin, M.: Light-induced pulsed EPR dipolar spectroscopy on a paradigmatic Hemeprotein, ChemPhysChem, 0, https://doi.org/10.1002/cphc.201900139, 2019.

Di Valentin, M., Albertini, M., Zurlo, E., Gobbo, M., and Carbonera, D.: Porphyrin Triplet State as a Potential Spin Label for Nanometer Distance Measurements by PELDOR Spectroscopy, J. Am. Chem. Soc., 136, 6582–6585, https://doi.org/10.1021/ja502615n, 2014.

Edwards, T. H. and Stoll, S.: Optimal Tikhonov regularization for DEER spectroscopy, J. Magn. Reson., 288, 58–68, https://doi.org/10.1016/j.jmr.2018.01.021, 2018.

Fábregas Ibáñez, L., Jeschke, G., and Stoll, S.: DeerLab: A comprehensive toolbox for analyzing dipolar EPR spectroscopy data, Magn. Reson. Discuss., 2020, 1–28, https://doi.org/10.5194/mr-2020-13, 2020.

Hintze, C., Bücker, D., Domingo Köhler, S., Jeschke, G., and Drescher, M.: Laser-Induced Magnetic Dipole Spectroscopy, J. Phys. Chem. Lett., 7, 2204–2209, https://doi.org/10.1021/acs.jpclett.6b00765, 2016.

Hogben, H. J., Krzystyniak, M., Charnock, G. T. P., Hore, P. J., and Kuprov, I.: Spinach – A software library for simulation of spin dynamics in large spin systems, J. Magn. Reson., 208, 179–194, https://doi.org/10.1016/j.jmr.2010.11.008, 2011.

Scherer, A., Yao, X., Qi, M., Wiedmaier, M., Godt, A., and Drescher, M.: Increasing the Modulation Depth of Gd(III)-Based Pulsed Dipolar EPR Spectroscopy (PDS) with Porphyrin-Gd(III) Laser-Induced Magnetic Dipole Spectroscopy, J. Phys. Chem. Lett., 13, 10958–10964, https://doi.org/10.1021/acs.jpclett.2c02138, 2022.

Stoll, S. and Schweiger, A.: EasySpin, a comprehensive software package for spectral simulation and analysis in EPR, J. Magn. Reson., 178, 42–55, https://doi.org/10.1016/j.jmr.2005.08.013, 2006.

Tait, C. E. and Stoll, S.: Coherent pump pulses in Double Electron Electron Resonance spectroscopy, Phys. Chem. Chem. Phys., 18, 18470–18485, https://doi.org/10.1039/C6CP03555H, 2016.

Williams, L., Tischlik, S., Scherer, A., Fischer, J. W. A., and Drescher, M.: Site-directed attachment of photoexcitable spin labels for light-induced pulsed dipolar spectroscopy, Chem. Commun., 56, 14669–14672, https://doi.org/10.1039/D0CC03101A, 2020.
